# Associations Between Declining Physical and Cognitive Functions in the Lothian Birth Cohort 1936

**DOI:** 10.1093/gerona/glaa023

**Published:** 2020-01-20

**Authors:** Judith A Okely, Ian J Deary

**Affiliations:** Lothian Birth Cohort Studies, Department of Psychology, University of Edinburgh, UK

**Keywords:** Common cause hypothesis, Longitudinal study, Grip strength, Processing speed

## Abstract

**Background:**

The ageing process is characterized by declines in physical and cognitive function. However, the relationship between these trajectories remains a topic of investigation.

**Methods:**

Using four data waves collected triennially between ages 70 and 79, we tested for associations between multiple cognitive ability domains (verbal memory, processing speed, and visuospatial ability) and physical functions (walking speed, grip strength, and lung function). We first tested for associations between linear declines in physical and cognitive functions over the entire 9-year study period, and then, for lead-lag coupling effects between 3-year changes in cognitive and physical functions.

**Results:**

Steeper linear decline in walking speed was moderately correlated with steeper linear declines in each cognitive domain. Steeper linear decline in grip strength was moderately correlated with steeper linear declines in verbal memory and processing speed. Lead-lag coupling models showed that decline in verbal memory was preceded by declines in walking speed and grip strength. By contrast, decline in grip strength was preceded by declines in processing speed and visuospatial ability, and decline in walking speed was preceded by decline in visuospatial ability. Following additional adjustment for covariates, only coupling effects from earlier decline in processing speed to later decline in grip strength remained significant (*β* = 0.545, *p* = .006).

**Conclusion:**

Our findings provide further evidence of an association between cognitive and physical declines and point to the potential order in which these changes occur. Decline in processing speed in particular may serve as a unique early marker of declining upper body strength.

The ageing process is characterized by declines in the mean levels of physical and cognitive function. An understanding of the dynamic association between these two domains is important for defining pathways to older age functional disability. However, the literature is inconclusive regarding the nature and degree of interdependence between cognitive and physical abilities and their respective rates of decline with ageing.

According to Cattell–Horn–Carroll (CHC) theory, cognitive abilities can be divided into broad domains which include crystallized ability (learned knowledge and experience), memory, visuospatial ability (mental representation and manipulation of visuospatial information), and processing speed (the time required to process information) (1). An alternative approach to describing cognitive function, based on neuropsychological theory, emphasizes the role of executive function (a higher-order process that controls and regulates basic cognitive functions). Whereas CHC theory is typically applied to the study of cognitive abilities in large nonpathological samples, executive function theory is mostly studied in clinical settings (2). The current study adopted the former CHC approach. Whereas crystallized ability remains relatively stable in its mean level throughout much of adult life, other cognitive domains tend to decline in mean level with age; processing speed follows a steady trajectory of decline originating in early adulthood; visuospatial and memory abilities typically start to decline in middle age (3,4).

Forced expiratory volume in 1 second (FEV_1_), grip strength, and walking speed are commonly used as objective indicators of physical function. Like memory and visuospatial ability, these physical functions, on average, peak in early adult life, begin to decline in midlife, and continue to decline in older age (5,6). Although declining cognitive and physical function is a near universal experience in older age, there is substantial variation between people in terms of levels of cognitive and physical function and their rates of decline with ageing (7,8).

According to the common cause hypothesis, a common physiological ageing process accounts for variance across basic sensory, physical, and cognitive functioning in old age, resulting in partly shared levels and trajectories of age-related decline in these functions (9,10). In support of this theory, some longitudinal studies find a small to moderate association between declines in cognitive and physical function (7,11,12). However, others report no such association (13,14). Inconsistent findings might indicate that this relationship varies as a function of the specific physical or cognitive domains under investigation. Indeed, studies that assessed multiple cognitive abilities indicate that processing speed and executive function domains are most consistently associated with physical functions (11,15,16).

Null findings have also prompted authors to consider an alternative account of the relationship between declining physical and cognitive health; specifically, that declines in cognitive and physical functions are related but that changes in these functions occur at different stages of the ageing process (7,12). Such a lead–lag relationship between declines in physical and cognitive functions could indicate that one domain is more sensitive to a “common cause” ageing processes than the other. Alternatively, such an effect might point to a causal relationship between declining cognitive and physical health.

Using multivariate latent change score modeling, it is possible to test for correlations between linear changes in different domains over the entire study period, and whether changes, between measurement occasions, in one domain predict subsequent changes in another (17). However, we are aware of only one study that has applied this model to the relationship between ageing physical and cognitive functions (18). This previous study found that earlier decline in gait speed predicted subsequent decline in cognitive function (indexed by the Digit Symbol Substitution Test and the Mini-Modified Mental State examination); however, the converse association (between earlier decline in cognitive function and later decline in gait speed) was weaker and nonsignificant.

The dearth of research examining potentially dynamic relationships between the ageing of cognitive and physical functions motivated the design of the present study. We build on previous work by: (a) testing for dynamic coupling effects between changes in physical and cognitive functions; (b) including three key indicators of physical function: FEV_1_, grip strength, and walking speed; (c) including three domains of cognitive ability: verbal memory, processing speed, and visuospatial ability (each indexed by multiple cognitive ability tests); (d) testing for associations in a narrow-age cohort and thus reducing the risk of confounding by chronological age (19); and (e) using data from four measurement occasions spanning the entire eighth decade of life.

## Methods

### Participants

The Lothian Birth Cohort 1936 (LBC1936) is a follow-up study of some people who took part in the Scottish Mental Survey of 1947 (SMS1947). This SMS1947 tested the mental ability of 70,805 Scottish school children born in 1936 at a mean age of 11 (20). Individuals born in 1936 and living in the Edinburgh and Lothians areas of Scotland, were contacted and recruited into the LBC1936 study. The first wave of the LBC1936 study was conducted between 2004 and 2007 with a sample of 1,091 participants (age mean [*M*] = 70, standard deviation [*SD*] = 0.83) (19,21,22). Since then, participants have returned for testing on a triennial basis, with waves 2, 3, and 4 taking place between 2007–2010 (*n* = 866; age *M* = 73, *SD* = 0.71), 2011–2013 (*n* = 697; age *M* = 76, *SD* = 0.68) and 2014–2017 (*n* = 550; age *M =* 79, *SD* = 0.62), respectively. Ethical approval was obtained from the Multi-Centre Ethics Committee for Scotland (MREC/01/0/56) and Lothian Research Ethics Committee (LREC/2003/2/29). All participants provided written informed consent.

### Measures

#### Cognitive ability

Participants completed the same battery of 13 cognitive tests at each wave of the study. These tests can be treated as indicators of four latent cognitive ability domains: visuospatial ability, processing speed, verbal memory, and crystalized ability (23). The Supplementary File details the cognitive tests that were used as indicators of the domains of visuospatial ability, processing speed, and verbal memory (we did not include the crystalized ability domain in the analysis). To allow consistent scaling, each cognitive test score at each wave was standardized by subtracting its mean score at wave 1, and dividing by its standard deviation at wave 1.

#### Physical function

Forced expiratory volume from the lungs in one second (FEV_1_), an indicator of lung function, was assessed using a Micro Medical Spirometer. Participants were given three attempts on the spirometer; we used the highest scoring attempt as the FEV_1_ variable. Grip strength in both hands was measured using a North Coast Hydraulic Hand Dynamometer. Participants performed this test three times with each hand. We used the highest grip score from all six attempts. The time (in seconds) to walk 6 m along a corridor was recorded with a stopwatch. In the results section, we refer to this variable as walking time; note that higher scores on this variable indicate a slower walking speed (ie, taking longer to walk 6 m). All the above tests were performed by nurses on the same day as participants completed cognitive testing.

#### Covariates

We included age, sex, age 11 IQ, height, and history of diabetes (type 1 or 2), cardiovascular disease, stroke, and hypertension as covariate variables. These variables have previously been associated with cognitive and physical abilities in older age (13,24–28). Age in days at time of testing was recorded at each wave. Age 11 IQ was assessed by the Moray House Test No. 12, which is described in detail elsewhere (20). Participants’ scores were corrected for age in days at time of testing. Height (in cm) was measured by a nurse on the same day as the physical and cognitive tests. Participants self-reported, at each wave, whether they had been diagnosed with diabetes, cardiovascular disease, stroke, or hypertension.

#### Analysis

We examined the longitudinal relationship between physical and cognitive function using an extension of the bivariate latent change score (LCS) model (17). See the Supplementary File for a description of the LCS framework, and details regarding the measurement model of each of the cognitive ability domains and physical functions.

**Figure 1.**
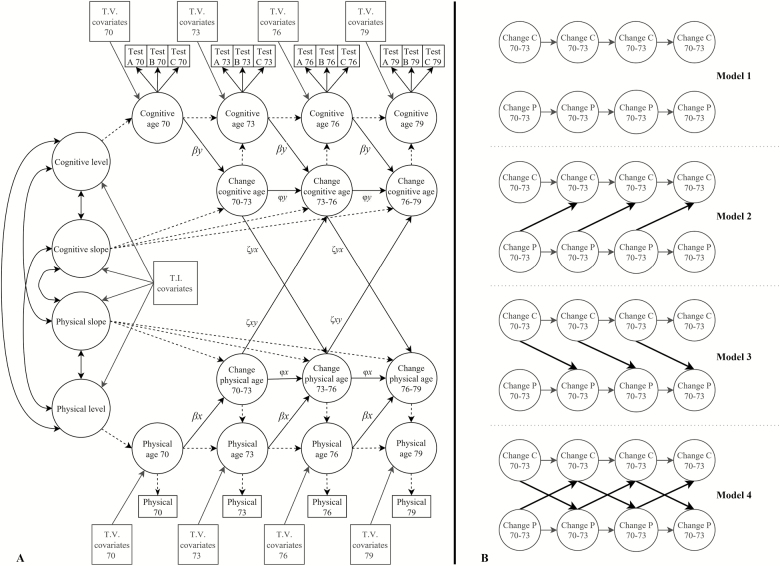
(**A**) Diagram of the bivariate latent change score model testing for dynamic coupling effects between changes in physical and cognitive functions. Single-headed arrows are regression effects and double headed arrows are covariances. Single-headed arrows with dashed lines show regression effects fixed at 1. Gray boxes show covariate variables; T.I. = time-invariant; T.V. = time-variant. Although not shown here, residuals of the same cognitive test were allowed to correlate across waves. (**B**) Simplified diagram of models 1–4. Arrows in black are dynamic coupling effects. Levels at each wave, autoregressive effects, intercepts, and slopes of cognitive and physical function not shown but were estimated in each model. C = cognitive ability, P = physical function. All other paths as described for panel A.

Applying the LCS framework, we estimated latent change scores representing reliable measures of change in physical and cognitive function between ages 70–73, 73–76, and 76–79; intercepts representing levels of physical and cognitive function at age 70; and slopes representing trend-like change in physical and cognitive function from age 70 to 79. Following the extension to this model proposed by Grimm, An, McArdle, Zonderman, and Resnick (17), we specified coupling effects from change in cognitive function to upcoming change in physical function (paths ζ*yx* in Figure 1A) and from change in physical function to upcoming change in cognitive function (paths ζ*xy* in Figure 1A). Auto-proportional effects for physical and cognitive function were also specified from the variable’s earlier level and change to its own upcoming change (paths *βx, βy, φx,* and *φy* in Figure 1A). Equality constraints (which force estimates to be equal over time) are often placed on auto-proportional and coupling parameters in LCS models; these constraints reflect the assumption that auto-proportional and coupling effects do not depend on the time span of the model. However, Grimm, Ram, and Estabrook (29) note that such equality constraints are not appropriate if the change process is dependent on time. As risk of declining physical and cognitive function increases with older age, we did not impose these equality constraints.

To examine associations between levels and trend-like change in physical and cognitive function, we first estimated a model with intercepts and slopes but no auto-proportional effects or dynamic coupling effects (this model is statistically equivalent to a bivariate growth curve model) (29). We refer to this model as model 0 in the results section. This model was firstly adjusted for sex and age at time of testing. To test whether other covariate variables might drive associations between intercepts and slopes of physical and cognitive function, the model was rerun additionally adjusting for age 11 IQ, height at time of testing, and history of chronic disease. Because the focus of this analysis was on intercept and slope correlations we adjusted for history of chronic disease (reporting a diagnosis of diabetes, stroke, cardiovascular disease, or hypertension at any wave of the study) at the intercept and slope level (rather than the true score level, as was done for models 1–4).

To test for time-dependent associations between changes in physical and cognitive function, we compared four possible models using likelihood-ratios tests of change in model fit (29). Model 1, which served as the baseline model, included auto-proportional effects but did not include coupling effects between changes in physical and cognitive function. Model 2 additionally included unidirectional paths from earlier change in physical function to later change in cognitive function. Model 3 tested the converse effect, and included unidirectional paths from earlier change in cognitive function to later change in physical function. If both models 2 and 3 resulted in improved fit over model 1, we ran model 4, which tested for bidirectional associations, and therefore included paths from earlier change in cognitive function to later change in physical function and, paths from earlier change in physical function to later change in cognitive function. Model 4 was compared to model 2 or 3, depending on which model provided the better fit. Models 1 to 4 are summarized in Figure 1B.

The two aims of this study were (a) to test for dynamic associations between declining physical and cognitive functions and (b) to test whether any such associations are accounted for by other ageing processes or individual differences. We therefore first ran the models, described above, correcting for age at time of testing and sex. The best fitting models were then rerun with additional correction for potentially mediating or confounding variables: height, age 11 IQ, history of hypertension, cardiovascular disease, stroke, and diabetes. Sex and age 11 IQ were treated as time-invariant predictors of levels and slopes of cognitive and physical function. Age, height, and history of chronic disease were recorded at each wave and treated as time-varying predictors of the true cognitive and physical function scores at each wave.

Given the large sample size and multiple significance tests in our analysis, we chose a significance threshold of *p* = .01 for parameter estimates and for testing changes in model fit. This alpha criterion has been applied in previous studies involving multiple significance tests or larger samples (30,31). Models were fit using all available data with full-information maximum-likelihood estimation (FIML). The bivariate latent change score models were fitted using Mplus Version 8 (32). We report both unstandardized and standardized parameter estimates below. Note that standardized estimates are standardized partial regression coefficients (as each change score is regressed on earlier levels and changes) and therefore are not confined to the bounds of (−1, 1) (33). Thus, standardized partial regression coefficients can indicate the strength of a predictor relative to other predictors in the model but do not indicate *objective* strength of prediction (as they are not bound within a specific range). Standardized estimates greater than one typically occur when predictor variables are correlated (ie, there is multicollinearity in the model) (33).

## Results

Table 1 shows characteristics of participants at each wave of the study. The Supplementary File provides details regarding participant attrition, trajectories of cognitive and physical test scores across the study, correlations between cognitive and physical test scores (within and between waves), and within-cognitive and within-physical level and slope correlations. We observed a moderate to strong correlation between slopes of cognitive functions, and a moderate correlation between slopes of FEV_1_ and grip strength but not between slopes of the other physical functions (see Supplementary Table 9).

**Table 1.** Characteristics of Participants at Each Wave of the Study

Variable	All Participants			
	Age 70	Age 73	Age 76	Age 79
*N*	1,091	866	697	550
Matrix reasoning	13.5 (5.1)	13.2 (5.0)	13.0 (4.9)	12.9 (5.0)
Block design	33.8 (10.3)	33.6 (10.1)	32.2 (10.0)	31.2 (9.6)
Spatial span	14.7 (2.8)	14.7 (2.8)	14.6 (2.7)	14.1 (2.7)
Verbal pairs	26.4 (9.1)	27.2 (9.5)	26.4 (9.6)	27.1 (9.6)
Logical memory	71.5 (18.0)	74.3 (17.9)	74.6 (19.2)	72.7 (20.4)
Digit span	7.7 (2.3)	7.8 (2.3)	7.8 (2.4)	7.6 (2.2)
Digit Symbol	56.6 (12.9)	56.4 (12.3)	53.8 (12.9)	51.2 (13.0)
Symbol search	24.7 (6.4)	24.6 (6.2)	24.6 (6.5)	22.7 (6.7)
Reaction time	0.6 (0.1)	0.70 (0.1)	0.70 (0.1)	0.70 (0.1)
Inspection time	112.1 (11.0)	111.2 (11.8)	110.1 (12.6)	107.0 (13.6)
FEV_1_	2.4 (0.7)	2.3 (0.7)	2.1 (0.6)	2.1 (0.6)
Grip strength	29.6 (10.2)	29.5 (9.4)	28.7 (10.0)	27.1 (9.4)
Walking time	3.9 (1.2)	4.4 (1.3)	4.7 (1.7)	5.2 (1.9)
Age	70 (0.8)	73 (0.7)	76 (0.7)	79 (0.6)
Diabetes	91 (8.3)	95 (11.0)	82 (11.8)	71 (13.0)
CVD	268 (24.6)	250 (28.9)	236 (33.9)	204 (37.2)
Hypertension	433 (39.7)	425 (49.1)	378 (54.3)	317 (57.6)
Height (in cm)	166.4 (8.9)	166.4(8.9)	165.9 (8.8)	165.3 (9.1)
Women	543 (49.8)			
Age 11 IQ	100 (15.0)			

*Note*: Data are shown as mean (*SD*) or *N* (%). CVD = cardiovascular disease; FEV_1_ = forced expiratory volume in 1 s.

### Intercepts and Linear Changes in Cognitive and Physical Function

In model 0, with no coupling effects and no auto-proportional effects, the unstandardized estimate of average linear change, in scaled cognitive test scores, per 3 years was −0.057, *p* < .001 for verbal memory; −0.166, *p* < .001 for processing speed; and −0.115, *p* < .001 for visuospatial ability. These linear changes varied significantly between individuals (*p* values for variance in changes in verbal memory, processing speed, and visuospatial ability were <.001, <.001, and .001, respectively). For the physical functions, the unstandardized linear change estimate (using raw scores) per 3 years was 0.576, *p* < .001 for walking time (note that this positive change indicates a decrease in walking speed); −1.06, *p* < .001 for grip strength; and −0.129 for FEV_1_*p* < .001. Linear change in walking time and grip strength varied significantly between individuals (*p* < .001) but linear change in FEV_1_ did not (*p =* .058). These estimates were taken from univariate models (which modeled changes in each domain of physical or cognitive function separately), but were similar to estimates in the bivariate models (which simultaneously modeled changes in physical and cognitive functions).

Table 2 shows age- and sex-adjusted and fully-adjusted estimates from bivariate models testing for associations between intercepts and linear changes (slopes) in physical and cognitive functions. Following adjustment for age and sex, there was a significant cross-sectional association between each level (intercept) of physical function at age 70 and each level of cognitive function at the same age, such that better physical function was associated with better cognitive function. Effect sizes ranged between *r* = .118 (for FEV_1_ and verbal memory) and *r* = −.391 (for walking time and processing speed). The strength of these intercept correlations was reduced following additional adjustment for age 11 IQ, height at time of testing, and history of chronic disease. Only associations between walking time and processing speed, grip strength and visuospatial ability, and FEV_1_ and processing speed remained statistically significant.

**Table 2.** Correlations Between Intercepts and Slopes of Physical and Cognitive Function

Physical	Cognitive	Intercepts				Slopes			
		Minimally Adjusted		Fully Adjusted		Minimally Adjusted		Fully Adjusted	
		*r*	*p*	*r*	*p*	*r*	*p*	*r*	*p*
Walking	Memory	**−.190**	<.001	.001	.992	**−.323**	<.001	**−.300**	<.001
Walking	Speed	**−.391**	<.001	**−.218**	<.001	**−.486**	<.001	**−.470**	<.001
Walking	Spatial	**−.307**	<.001	**−**.131	.013	**−.430**	<.001	**−.470**	<.001
Grip	Memory	**.129**	.002	.009	.862	**.391**	<.001	**.374**	<.001
Grip	Speed	**.251**	<.001	.100	.019	**.414**	<.001	**.408**	<.001
Grip	Spatial	**.287**	<.001	**.185**	<.001	.393	.032	.356	.053
FEV_1_	Memory	**.118**	.003	**−**.030	.532	.318	.092	.413	.044
FEV_1_	Speed	**.313**	<.001	**.151**	<.001	.396	.044	.478	.022
FEV_1_	Spatial	**.253**	<.001	.108	.011	**−**.027	.924	**−**.092	.754

*Note:* Estimates from model 0 (with no coupling effects and no auto-proportional effects). Estimates in bold are statistically significant. Minimally adjusted estimates are adjusted for sex and age at time of testing; fully-adjusted estimates are additionally adjusted for age 11 IQ, height at time of testing, and history of chronic disease (ever diagnosed with diabetes, stroke, cardiovascular disease, or hypertension). Memory = verbal memory, speed = processing speed, spatial = visuospatial ability. FEV_1_ = forced expiratory volume in 1 s.

Following adjustment for age and sex, linear change in walking time was significantly correlated with linear change in verbal memory (*r* = −.323, *p* < .001), processing speed (*r* = −.486, *p* < .001), and visuospatial ability (*r* = −.430, *p* <.001). Linear change in grip strength was significantly correlated with linear change in verbal memory (*r* = .391, *p* < .001) and processing speed (*r* = .414, *p* < .001), but not visuospatial ability. Linear change in FEV_1_ was not significantly correlated with linear change in any of the cognitive ability domains. Statistically significant correlations between linear slopes were all in the same direction: individuals who experienced a steeper decline in physical function were likely to also experience a steeper decline in cognitive function. Each of the statistically significant slope correlations survived adjustment for potential covariates (age 11 IQ, height at time of testing, and history of chronic disease). The effect sizes of these correlations were, on average, only slightly reduced in the fully-adjusted model. See the Supplementary File and Supplementary Table 10 for details regarding correlations between intercepts of cognitive function and slopes of physical function, and vice versa.

**Figure 2.**
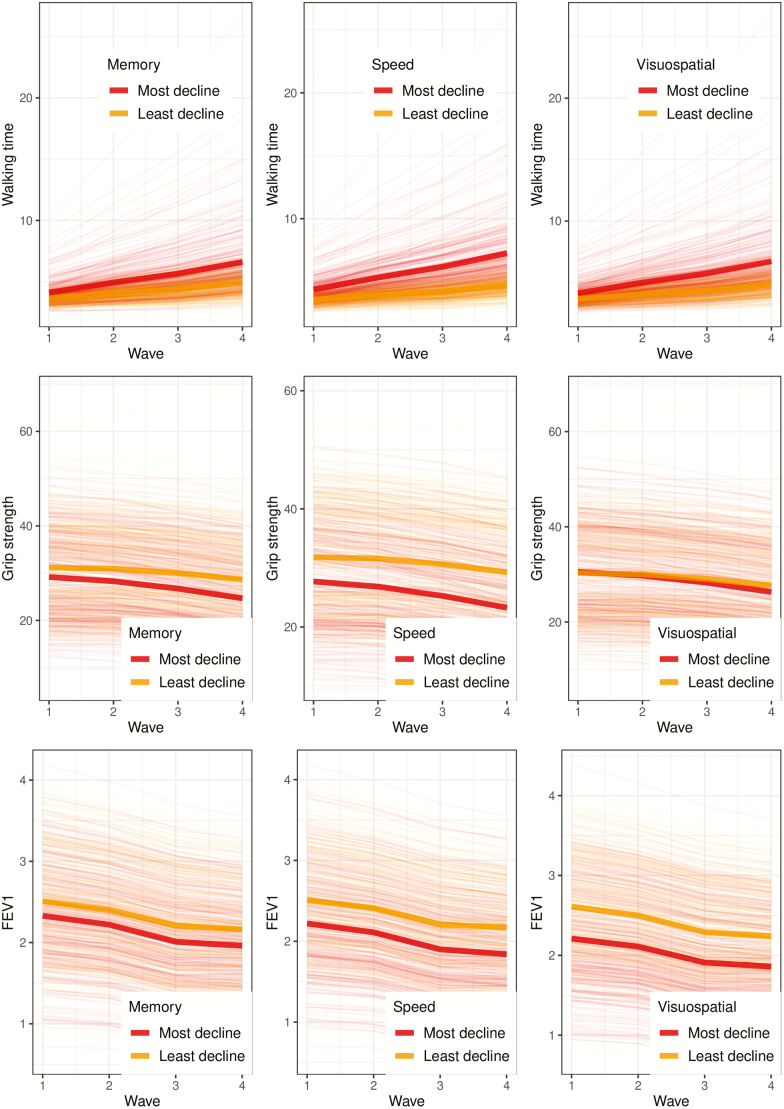
Individual trajectory plots of physical function for participants in the top and bottom quartile of linear cognitive decline over the duration of the study. Physical function trajectories in red/dark gray show participants who experienced the most cognitive decline, and physical function trajectories in yellow/light gray show participants who experienced the least cognitive decline. Bold red/dark gray and yellow/light gray lines show the mean trajectory for the most and least cognitive decline groups respectively. Physical function scores were adjusted for age and sex and were estimated for all participants under full-information maximum-likelihood estimation (FIML).

The relationship between linear change in physical and cognitive function is illustrated in Figure 2, which shows individual trajectories of physical function grouped according to top and bottom quartiles of cognitive decline. Model 0 specifies a linear pattern of change in physical and cognitive function and serves as a baseline model. However, it is possible that changes in these variables are nonlinear. Supplementary Table 11 shows correlations between concurrent change scores estimated in model 1 (which specifies exponential changes in physical and cognitive functions over time).

Estimates from model 0 indicated that linear changes in FEV1 did not vary significantly between individuals and that linear changes in FEV_1_ did not correlate with linear changes in cognitive functions. Owing to the low between-person variance in FEV_1_ change, we did not test for dynamic coupling effects between changes in cognitive functions and FEV_1_ (attempts to do so resulted in model nonconvergence or out of bounds estimates in most cases).

### Dynamic Coupling Effects Between Changes in Physical and Cognitive Function

Next, we tested for dynamic coupling effects between changes in physical and cognitive function by comparing fit indices for models 1, 2, 3, and 4 (which are described in the methods section and shown in Figure 1B).

Below, we describe results from model comparisons for each physical function (walking time and grip strength) in combination with each cognitive function (verbal memory, processing speed, and visuospatial ability) in turn. Supplementary Table 12 shows fit indices for models 1–4 for each physical and cognitive function combination and Table 3 shows standardized and unstandardized coupling estimates from the best fitting models. Coefficients are interpreted as the influence of dynamic change coupling effects after controlling for auto-proportional change processes and covariate variables.

**Table 3.** Coupling Estimates from the Best Fitting Models

Path	Age and Sex-Adjusted Estimates				Fully-Adjusted Estimates			
	*β*	B	B 99% CI	*p*	*β*	B	B 99% CI	*p*
*Walking time and verbal memory*								
∆ walk 70–73 →∆ memory 73–76	−0.334	−0.334	−0.766, 0.097	.046	0.316	0.307	−0.123, 0.738	.066
∆ walk 73–76 →∆ memory 76–79	−**0.611**	−**0.250**	−**0.405,** −**0.094**	**<**.001	−0.101	−0.028	−0.179, 0.123	.634
*Walking time and visuospatial ability*								
∆ visuospatial 70–73 →∆ walk 73–76	0.085	2.036	−14.214, 18.288	.747	1.268	12.737	−27.509, 52.984	.415
∆ visuospatial 73–76 →∆ walk 76–79	−1.582	−30.328	−62.462, 1.806	.015	−0.967	−111.992	−825.061, 601.076	.686
*Grip strength and verbal memory*								
∆ grip 70–73 →∆ memory 73–76	**0.662**	**0.304**	**0.059, 0.548**	.001	0.048	0.014^a^	−0.144, 0.172	.819
∆ grip 73–76 →∆ memory 76–79	**0.976**	**0.097**	**0.030, 0.165**	<.001	0.489	0.029^a^	−0.014, 0.073	.082
*Grip strength and processing speed*								
∆ speed 70–73 →∆ grip 73–76	0.359	15.433	−7.046, 37.912	.077	0.095	2.902	−19.965, 25.768	.744
∆ speed 73–76 →∆ grip 76–79	**0.642**	**7.703**	**2.638, 12.767**	**<**.001	**0.545**	**5.538**	**0.345, 10.730**	.006
*Grip strength and visuospatial ability*								
∆ spatial 70–73 →∆ grip 73–76	0.730	53.54	−10.18, 117.25	.030	0.346	1.574 ^ab^	−4.085, 7.233	.474
∆ spatial 73–76 →∆ grip 76–79	**0.965**	**29.45**	**8.87, 50.04**	**<**.001	0.857	1.887 ^ab^	−0.355, 4.129	.030

*Note*: ∆ = change, *β* = standardized estimate, B = unstandardized estimate. 99% CI and *p* values shown for unstandardized estimates. Estimates in bold are statistically significant. Fully-adjusted estimates are additionally adjusted for age 11 IQ, height and history of chronic disease.

^a^Model not adjusted for hypertension. ^b^Grip strength score at each wave divided by 10.

### Walking Time

#### Verbal memory

Likelihood ratio tests showed that model 2 (with unidirectional coupling from change in walking time to upcoming change in verbal memory; Figure 1) was the best fitting model (^∆^X^2^ = −17, *p* < .001). Parameter estimates from this model are shown in Supplementary Table 13. In model 2, auto-proportional effects from earlier levels or changes to upcoming changes were nonsignificant for walking time and for verbal memory. The first coupling estimate from change in walking time between ages 70 and 73 to upcoming 3-year change in verbal memory was B = −0.334, *p =* .046; *β* = −0.334; the second coupling estimate from change in walking time between ages 73 and 76 to upcoming 3-year change in verbal memory was B = −0.250, *p* < .001; *β* = −0.611. This latter significant effect indicates that greater increase in walking time between ages 73 and 76 was related to steeper subsequent decline in verbal memory.

#### Processing speed

Likelihood ratio tests showed the no coupling model (model 1) provided the best fit to the data. This result suggests that, although linear changes in walking time and processing speed are correlated (as shown in Table 2 and Figure 2), there is no lead–lag relationship between those changes. Parameter estimates from model 1 are shown in Supplementary Table 14, auto-proportional effects from earlier levels to upcoming changes were nonsignificant for walking time and for processing speed. Auto-proportional effects from earlier changes to upcoming changes were nonsignificant for walking time but were significant for processing speed. Steeper earlier decline in processing speed, between ages 70 and 73 or between ages 73 and 76, was related to steeper subsequent 3-year decline in processing speed—suggesting an accelerated decline in processing speed.

#### Visuospatial ability

Likelihood ratio tests showed that model 3 (with unidirectional coupling from change in visuospatial ability to upcoming change in walking time; Figure 1) was the best fitting model (^∆^X^2^ = −20, *p* < .001). Parameter estimates from this model are shown in Supplementary Table 15. In model 3, auto-proportional effects from earlier levels or changes to upcoming changes were nonsignificant for walking time and for visuospatial ability. The first coupling estimate from change in visuospatial ability between ages 70 and 73 to upcoming 3-year change in walking time was B = 2.036, *p* = .747; *β* = 0.085; the second coupling estimate from change in visuospatial ability between ages 73 and 76 to upcoming 3-year change in walking time was B = −30.328, *p* = .015; *β* = −1.582. This latter effect, which was close to the chosen significance level, suggests that a steeper decline in visuospatial ability between ages 73 and 76, was related to greater subsequent increase in walking time.

### Grip Strength

#### Verbal memory

Likelihood ratio tests showed that both model 2 (unidirectional paths from change in grip strength to upcoming change in verbal memory; Figure 1) and model 3 (unidirectional paths from change in verbal memory to upcoming change in grip strength) significantly improved model fit compared to model 1 (no dynamic coupling effects), and that model 2 had better fit than model 3. The full coupling model (model 4) did not result in better fit than model 2. Therefore, model 2 was the best fitting model (^∆^X^2^ = −26, *p* < .001). Parameter estimates from model 2 are shown in Supplementary Table 16. In this model, the auto-proportional effect from memory at age 73 to upcoming change in memory between ages 73 and 76 was negative and significant, indicating that a higher level of memory at age 73 was related to steeper 3-year decline. The remaining auto-proportional effects for memory (from levels and changes to upcoming changes) were nonsignificant. Auto-proportional effects for grip strength, from earlier levels to upcoming changes were nonsignificant. The auto-proportional effect from change in grip strength between ages 73 and 76 to upcoming 3-year change in grip strength was negative and significant, indicating that a steeper decline between ages 73 and 76 was related to less decline over the following 3 years. The coupling effect from change in grip strength between ages 70 and 73 to upcoming 3-year change in verbal memory was significant (B = 0.304, *p* = .001; *β* = 0.662), as was the coupling effect from change in grip strength between ages 73 and 76 to upcoming 3-year change in verbal memory (B = 0.097, *p* < .001; *β* = 0.976). These effects show that steeper declines in grip strength were related to subsequent steeper declines in verbal memory, and, that these associations became slightly stronger with increasing age.

#### Processing speed

Likelihood ratio tests showed that model 3 (with unidirectional coupling from change in processing speed to upcoming change in grip strength; Figure 1) was the best fitting model (^∆^X^2^ = −20, *p* < .001). Parameter estimates from model 3 are shown in Supplementary Table 17. Auto-proportional effects from level to upcoming change were nonsignificant for processing speed and for grip strength. However, auto-proportional effects from earlier change in processing speed to upcoming change in processing speed were positive and significant, indicating that steeper earlier decline predicted steeper subsequent decline. Auto-proportional effects from earlier changes in grip strength to upcoming changes in grip strength were also significant. Steeper decline in grip strength between ages 70 and 73 was related to steeper decline in grip strength between ages 73 and 76; however, steeper decline in grip strength between ages 73 and 76 was related to less decline in grip strength over the final interval of the study, between ages 76 and 79. The coupling estimate from change in processing speed between ages 70 and 73 to upcoming change in grip strength between ages 73 and 76 was B = 15.433*, p* < .077; *β* = 0.359; the coupling estimate from change in processing speed between ages 73 and 76 to upcoming 3-year change in grip strength was B = 7.703, *p* < .001; *β* = 0.642. This latter significant effect shows that steeper decline in processing speed between ages 73 and 76 was related to steeper subsequent decline in grip strength.

#### Visuospatial ability

Likelihood ratio tests showed that model 3 (with unidirectional coupling from change in visuospatial ability to upcoming change in grip strength; Figure 1) was the best fitting model (^∆^X^2^ = −36, *p* < .001). Parameter estimates from model 3 are shown in Supplementary Table 18. In model 3, auto-proportional effects from earlier levels or changes of visuospatial ability to upcoming change in visuospatial ability were nonsignificant. Auto-proportional effects from earlier levels of grip strength to upcoming changes in grip strength were also nonsignificant. However, steeper decline in grip strength between ages 70 and 73 was related to steeper subsequent decline in grip strength between ages 73 and 76. The coupling effect from change in processing speed between ages 70 and 73 to upcoming 3-year change in grip strength was B = 53.54, *p* = .030; *β* = 0.730. The coupling effect from change in visuospatial ability between ages 73 and 76 to upcoming 3-year change in grip strength was B = 29.45, *p* < .001; *β* = 0.965. This latter significant effect shows that steeper decline in visuospatial ability between ages 73 and 76 was related to steeper subsequent decline in grip strength.

### Adjustment for Covariate Variables

**Figure 3.**
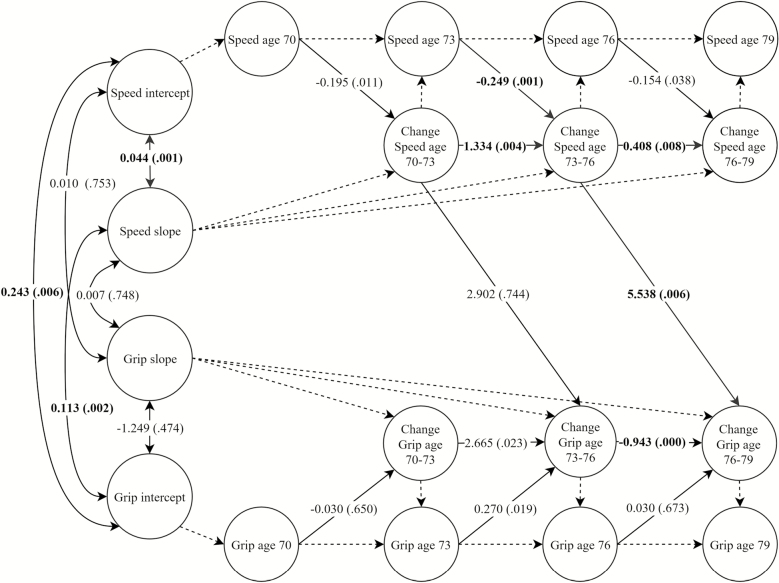
Simplified diagram of the fully-adjusted model of grip strength and processing speed (model 3). Estimates are unstandardized auto-proportional and coupling effects (single-headed arrows) and unstandardized covariances (double-headed arrows). Numbers in parentheses are *p* values. Estimates in bold are statistically significant. Arrows with dashed lines show regression effects fixed at 1.

Table 3 displays coupling effects from the best fitting models following additional adjustment for height, age 11 IQ, and history of chronic disease (diabetes, stroke, cardiovascular disease, and hypertension). The only coupling effect to survive additional adjustment for these covariates was from change in processing speed between ages 73 and 76 to upcoming 3-year change in grip strength (B = 5.538, *p* = .006; *β* = 0.545). Associations between covariate variables and physical and cognitive functions are shown in Supplementary Tables 19 and 20. Parameter estimates from the fully-adjusted model of processing speed and grip strength are shown in Figure 3. Owing to the complexity of the fully-adjusted analyses, some changes were required to allow certain fully-adjusted models to converge on within bounds estimates. These changes are detailed in the Supplementary File.

### Subsidiary Analysis

An alternative specification of the bivariate latent change score model includes lead-lag coupling effects from earlier levels to upcoming changes (rather than from earlier changes to upcoming changes, as specified in our analysis). In subsidiary analysis, model comparisons (of models 1–4) were rerun additionally controlling for the effect of earlier levels of physical function on upcoming changes in cognitive function. See the Supplementary File and Supplementary Tables 21 and 22 for further details. In contrast with our main results, baseline models (model 1) of verbal memory and walking time and verbal memory and grip strength were not improved by the addition of lead–lag coupling effects between changes in physical and cognitive functions (models 2 or 3). However, in line with our main results, model 3 of processing speed and grip strength (paths from earlier changes in processing speed to upcoming changes in grip strength) was the best fitting model.

## Discussion

The interdependence between cognitive and physical trajectories with ageing remains a key topic of investigation with important implications for the timing of interventions to support healthy ageing. We examined the relationship between parallel and dynamic time-ordered changes in physical and cognitive functions across the eighth decade of life. We found that (a) 9-year declines in walking speed were paralleled by declines in each cognitive ability (verbal memory, processing speed, and grip strength) over the same time period. (b) Nine-year declines in grip strength were paralleled by declines in verbal memory and processing speed (but not visuospatial ability). (c) Steeper 3-year declines in visuospatial ability predicted steeper subsequent declines in grip strength and walking speed. (d) Steeper 3-year declines in processing speed predicted steeper subsequent declines in grip strength. (e) Steeper 3-year declines in walking speed and grip strength predicted steeper subsequent declines in verbal memory. (f) These lead-lag coupling effects were stronger at later waves of the study. (g) Only the coupling effect from earlier decline in processing speed to later decline in grip strength survived correction for other potential predictors of physical or cognitive decline (premorbid cognitive ability, height, and history of chronic disease).

Our finding of a moderate correlation between declining physical functions (walking speed and grip strength) and cognitive functions (verbal memory, processing speed, and visuospatial ability) corroborates some previous reports (7,11,12) and provides further evidence in support of a “common cause” account of ageing (10). In our study, declines in cognitive abilities were not significantly correlated with decline in lung function (as indexed by FEV_1_); however, this null result may reflect the fact that, in our sample, slopes for FEV_1_ did not vary significantly between participants. It is notable that another study (13), using LBC1936 data, into changes in physical functions and fluid intelligence between age 70 and 76 (ie, three waves of data rather than the four waves available here), found a correlation between change in fluid intelligence and change in walking speed, *r* = .244, *p* = .039, but not between change in fluid intelligence and change in FEV_1_ or grip strength. The fact that we observed stronger correlations between changes in cognitive and physical functions suggests that these relationships may become stronger with older age.

One previous study tested for time-ordered associations between changes in physical and cognitive functions (18). Best et al. (18) found a unidirectional path from earlier decline in gait speed to subsequent decline in cognitive function. Our finding of the opposite direction of effect in the case of processing speed and visuospatial ability contrasts with those previous results. It is possible that the more comprehensive measures of processing speed and visuospatial ability in the present study were more sensitive to age-related changes, potentially detecting changes occurring earlier in the ageing process. In addition, the study by Best et al. (18) involved a longer interval between measurement occasions (4 or 5 years) and participants with a wider age range (between 70 and 79 years at baseline). Nevertheless, our finding of a path from earlier decline in walking speed or grip strength to later decline verbal memory is consistent with the sequence of changes described by Best et al. (18).

We note that other studies also tested for potential bidirectional associations between physical and cognitive functions over time. Findings have been mixed, with several studies reporting a unidirectional path from physical function to subsequent cognitive function (34–36), others reporting the opposite direction of effect, from cognitive function to subsequent physical function (37–39), and still others finding evidence of a bidirectional relationship between physical and cognitive functions over time (40–42). However, owing to methodological differences, results from these studies are not directly comparable to those described here. Many of these studies applied statistical models that test whether levels in one function predict subsequent change in the other. It is commonly assumed that low levels of physical or cognitive function in older age indicate greater age-related decline; however, low performance on these measures might result from long-standing individual differences originating earlier in life. Others applied cross-lagged panel models to investigate lead-lag associations between changes in cognitive and physical functions over multiple assessment occasions (41–43). However, a limitation of these models is that they describe change at the between-person level. That is, whether an individual’s rank order (how they rank relative to others on a particular measure) changes over time; these parameters can fail to represent within-person change, particularly when stable trait-like variables (such as cognitive or physical function) are used (44).

Overall, our findings support the hypothesis that associations between age-related changes in physical and cognitive functions follow a time-ordered sequence, with declines in visuospatial ability and processing speed generally preceding decline in physical function, and declines in physical function preceding declines in verbal memory. Such a cascade of events may reflect the effect of a common cause ageing process which potentially impacts rates of decline across physical and cognitive domains at different stages. Our results could show that declines in processing speed and visuospatial ability serve as early markers of these ageing processes which later impact other functions. There is evidence from previous investigations that processing speed, in particular, can serve as an early predictor of generalized decline in cognitive functioning (45). Our findings could suggest that declines in processing speed (and possibly visuospatial ability) also herald upcoming declines in physical function.

Following adjustment for potentially confounding or mediating variables (childhood cognitive ability, height, and history of diabetes, cardiovascular disease, stroke, or hypertension), or levels of physical function on upcoming change in cognitive function (in subsidiary analysis), only the path from earlier decline in processing speed to later decline in grip strength remained significant. This result suggests that the remaining coupling effects (from declines in visuospatial ability to subsequent declines in walking speed and grip strength, and from declines in walking speed and grip strength to subsequent declines in verbal memory) were at least partly driven by differences in physical function, health variables, or premorbid cognitive ability. The surviving link between processing speed and grip strength is comparable to reports from some previous studies. Two studies that assessed various domains of cognitive function (15,16) found a stronger association between physical functions and processing speed and weaker or nonsignificant associations with verbal memory, following adjustment for various covariate variables. In addition, grip strength, which can serve as a marker of central nervous system integrity, was found to be most consistently associated with cognitive function when compared with walking speed and sit-to-stand transfers in a study of older women (37). The authors of that study suggest that grip strength is more sensitive to age-related changes than are other measures of physical function. It is also possible that associations between declines in processing speed and grip strength are simply driven by declining motor skills. Performance on tests of processing speed (eg, inspection time and four choice reaction time) depends, to some extent, on hand motor skills which may also be related to grip strength.

A final notable finding of the present study is that declines in physical and cognitive function became more closely related with increasing age. This phenomenon is consistent with some previous reports (9,11,36) and with the description of a critical period in later-life when age-related changes begin to impact a broad spectrum of bodily functions (9).

Advantages of the present study include the narrow-age range of the cohort, the relatively long follow-up period (age 70–79 years) and that each cognitive domain was assessed using multiple tests. Methodological limitations include the initial composition of the cohort (healthy, community dwelling older people), and selective attrition over the follow-up (related to poorer performance on the physical and cognitive tests). These factors may have resulted in an underestimate of declines in physical and cognitive function that occur in the general population, and, potentially, the relationship between those declines. Furthermore, participants were all Caucasian and drawn from a limited geographical area; thus, replication of this study in a more diverse population of older people is warranted. The sample size (1,091 at baseline and 550 at the final wave of follow-up) is smaller than samples used by some other studies into physical and cognitive function. It is possible that our analysis was under-powered. However, the physical and cognitive function measures had good measurement properties and were assessed on several occasions (both factors that increase power of latent change score models (17)). We lowered the significance threshold to *p* = .01 to account for the multiple significance test in our analysis. However, we acknowledge that other more conservative approaches (eg, applying a correction across all *p*-values in the model) are possible. Thus, the significant results reported here should be interpreted cautiously and ideally replicated. Age, height, and history of chronic disease were recorded at each wave of the study and treated as time-varying predictors of cognitive and physical function levels at each wave. However, we did not test whether changes in those covariate variables accounted for changes in physical or cognitive function directly. Although such processes could be modeled within the latent change score framework, it would introduce considerable statistical complexity. In order to maintain model parsimony, we did not adopt this approach here. However, we recommend that future studies further explore the relationship between changing health processes in relation to changing physical and cognitive functions. We also note that physical and cognitive functions were assessed on a 3-yearly basis; therefore, potential associations between changes occurring over shorter time intervals were not captured.

## Conclusion

Our results provide further evidence of a relationship between declining physical and cognitive functions with ageing, and help to map out the order in which those declines occur. Results from the fully-adjusted models suggest that declining processing speed in particular may serve as a unique early marker of declining grip strength.

## Supplementary Material

glaa023_suppl_Supplementary_Material
