# Supplementary material for: Associations Between Declining Physical and Cognitive Functions in the Lothian Birth Cohort 1936
Source: J Gerontol A Biol Sci Med Sci. 2020 Jan 20;75(7):1393–402. doi: 10.1093/gerona/glaa023 (PMC7447860; doi:10.1093/gerona/glaa023)
Supplement: glaa023_suppl_Supplementary_Material [file glaa023_suppl_supplementary_material.docx]

**Supplementary Material**

**Methods**

*Cognitive Ability Tests*

Scores on the Spatial Span (Forward and Backward) subtest from the Wechsler Memory Scale, 3rd UK Edition (1), and the Matrix Reasoning and Block Design subtests from the from the Wechsler Adult Intelligence Scale, 3rd UK Edition (2) were used as indicators of visuospatial ability. Scores on the Symbol Search and Digit-Symbol Substitution tests from the Wechsler Adult Intelligence Scale, 3rd UK Edition (2), a computer-based inspection time test (3) and a four-choice reaction time test (4) were used as indicators of processing speed. Scores on the Digit Span Backward subtest from the Wechsler Adult Intelligence Scale, 3rd UK Edition (2), and the Verbal Paired Associates and Logical Memory subtests from the Wechsler Memory Scale, 3rd UK Edition (1) were treated as indicators of verbal memory. We did not include the crystalized ability domain in the current analysis.

*Elements of the Bivariate Latent Change Score (LCS) Model*

Bivariate LCS models combine elements of cross-lag models, which estimate lead-lag coupling effects between variables across consecutive time points, and latent growth curve models, which estimate intercepts (initial levels) and slopes (describing each person’s growth trend across all time points in the study). LCS models improve on more traditional cross-lag models which fail to account for trend-like change – a limitation which can lead to spurious results (5). Panel A of Figure 1 of the paper shows the LCS model used in the current analysis. Model parameters include latent change scores which represent reliable measures of change between consecutive measurement occasions (these are labelled “change cognitive” and “change physical” in Figure 1); intercepts which represent initial levels of each variable at the first measurement occasion (these are labelled “physical intercept” and “cognitive intercept” in Figure 1); and constant change components or slopes which represent trend-like change over the duration of the study (these are labelled “physical slope” and “cognitive slope” in Figure 1). Whereas latent change scores represent change between consecutive measurement occasions, slopes represent a person’s growth trend across the entire study, across all measurement occasions. Paths from a variable’s earlier level and latent change score to its own upcoming latent change score are called auto-proportional effects (in Figure 1, auto-proportional effects from a variable’s level to its own upcoming latent change score are labelled *βx* and *βy;* auto-proportional effects from a variable’s earlier latent change score to its own upcoming latent change score are labelled *φx* and *φy*)*.* Paths between two different variables representing lead-lag associations between earlier changes in one variable and subsequent changes in the other are termed coupling effects, these are labelled ζ*xy* and ζ*yx* in Figure 1, and are the key paths of interest in the present study.

*Measurement Model*

Each of the three cognitive ability domains were modeled as latent variables with scores on three or four (in the case of processing speed) cognitive tests serving as indicators. Latent factors were scaled using the marker variable method, with the first cognitive test loading set to 1. Strong measurement invariance was established by imposing equality constraints on the loadings and intercepts of each (remaining) repeated cognitive ability tests. Residuals of the same test were allowed to correlate across 3- 6- and 9-year intervals. Residual correlations over the same time lag (i.e., repeated 3- or 6-year intervals) were set to be equal. A previous growth curve modelling study, with three waves of LBC1936 data, found that changes in physical functions (FEV_1_, grip strength, and walking time) were not correlated (6). Using the same growth curve modelling approach, but now with four waves of data, and controlling for sex, height (at time of testing) and age (at time of testing), we found a significant correlation between slopes of FEV_1_ and grip strength (*r* = 0.557, *p* < 0.002), but not between slopes of FEV_1_ and walking time (*r* = -0.111, *p* = 0.361), or slopes of walking time and grip strength (*r* = -0.168, *p =* 0.093). See supplementary Table 9. Because not all physical declines were correlated, we chose to model change in each function separately. At each wave, each physical function was modelled as a latent true score indicated by the observed physical function score and an error term. A separate model was run for each of the physical functions testing for associations with each cognitive ability domain (verbal memory, processing speed and visuospatial ability) in turn.

**Results**

*Sample Characteristics*

We report characteristics of the sample at each wave (under ‘all participants’) in Table 1 of the paper and of participants who attended all four waves of the study (under ‘completers’) in Supplementary Table 1 (N = 539). Mean performance on most of the cognitive tests decreased over the four waves of testing among ‘all participants’ and ‘completers’. However, mean scores on the verbal pairs and logical memory tests did not decrease when looking across ‘all participants’. FEV_1_ and Grip strength decreased over time, and the time to walk 6m increased. Individual trajectories in physical and cognitive functions across the four waves are shown in Supplementary Figure 1. The proportion of participants reporting a history of diabetes, cardiovascular disease, stroke and hypertension increased over the four waves. The mean height of participants decreased from age 73 onwards among ‘all participants’ and from age 70 onwards among ‘completers’. Correlations between height measures at consecutive waves of the study ranged between *r* = 0.985 (between ages 70 and 73) and *r* = 0.989 (between ages 76 and 79).

Supplementary Table 2 shows differences, at age 70, between participants who provided data at all waves (‘completers’) and participants who provided data at fewer than 4 waves (‘non-completers’). Completers performed better than non-completers on the physical tests and achieved higher scores on all of the cognitive tests. Completers were, on average, taller than non-completers and were less likely to report a history of diabetes, stroke or hypertension.

| **Supplementary Table 1.** Sample Characteristics of Completers at Each Wave of the Study | | | | |
| --- | --- | --- | --- | --- |
|  | Completers | | | |
|  | Age 70 | Age 73 | Age 76 | Age 79 |
| Matrix reasoning | 14.5 (5.0) | 13.9 (4.9) | 13.3 (4.9) | 13.0 (5.0) |
| Block design | 35.7(10.1) | 34.7 (10.1) | 32.7 (9.8) | 31.3 (9.7) |
| Spatial span | 15.1 (2.8) | 14.9 (2.7) | 14.8 (2.7) | 14.2 (2.7) |
| Verbal pairs | 27.9 (8.5) | 28.5 (9.0) | 27.3 (9.3) | 27.3 (9.4) |
| Memory | 74.4 (16.9) | 76.3 (16.9) | 75.8 (18.5) | 73.0 (20.3) |
| Digit span | 8.0 (2.4) | 8.0 (2.3) | 7.9 (2.4) | 7.6 (2.2) |
| Digit Symbol | 59.0 (12.4) | 58.4 (12.0) | 55.4 (12.3) | 51.4 (13.0) |
| Symbol search | 25.6 (6.6) | 25.5 (5.9) | 25.3 (6.3) | 22.7 (6.7) |
| Reaction time | 0.6 (0.1) | 0.6 (0.1) | 0.7 (0.1) | 0.7 (0.1) |
| Inspection time | 113.4 (10.5) | 112.3 (11.7) | 111.0 (11.8) | 107.1 (13.6) |
| FEV_1_ | 2.5 (0.7) | 2.4 (0.7) | 2.2 (0.6) | 2.1 (0.6) |
| Grip strength | 30.3 (10.2) | 30.0 (9.5) | 28.8 (9.6) | 27.2 (9.4) |
| Walking time | 3.7 (1.0) | 4.2 (1.1) | 4.6 (1.5) | 5.2 (1.9) |
| Age | 70 (0.8) | 73 (0.7) | 76 (0.7) | 79 (0.6) |
| Diabetes | 35 (6.5) | 44 (8.2) | 59 (11.0) | 69 (12.9) |
| CVD | 123 (22.8) | 151 (28.0) | 185 (34.3) | 198 (36.8) |
| Stroke | 18 (3.3) | 31 (5.8) | 55 (10.2) | 67 (12.5) |
| Hypertension | 193 (35.8) | 256 (47.5) | 286 (53.1) | 314 (58.3) |
| Height (in cm) | 167.1 (8.9) | 166.6 (8.9) | 166.1 (8.9) | 165.4 (9.1) |
| Women | 267 (49.5) |  |  |  |
| Age 11 IQ | 102 (15.2) |  |  |  |
| *Note.* Data are shown as mean (SD) or N (%). | | | | |


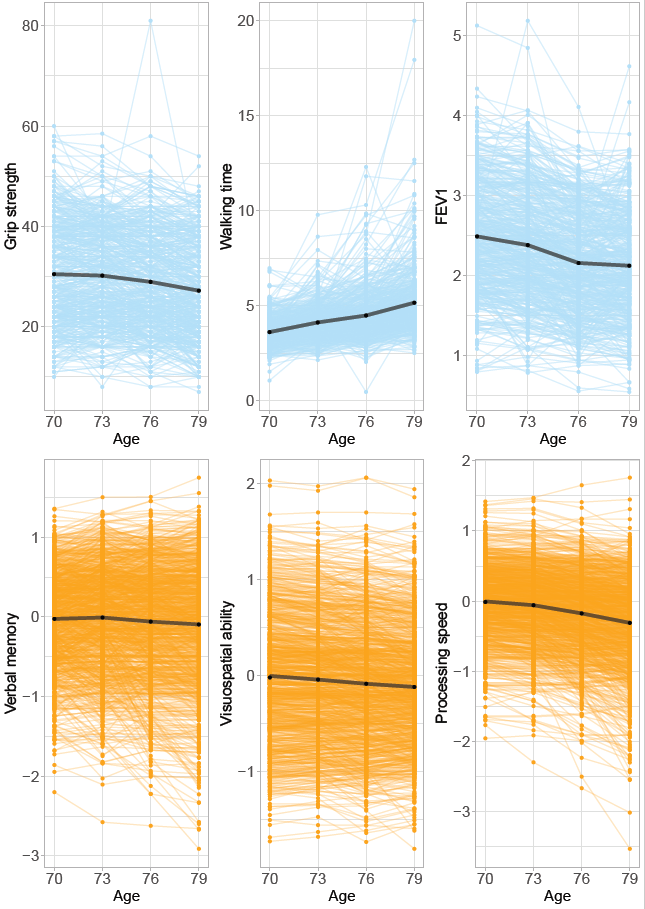


**Supplementary Figure 1.** Individual trajectory plots of physical and cognitive function at each wave of the study (at mean ages 70, 73, 76, and 79). Black dots represent mean levels at each wave. Grey lines show the smoothed conditional mean trajectory. Note that higher scores on walking time indicate poorer performance. Trajectory plots for physical functions only show data for participants attending all four waves. Factor scores for cognitive functions were estimated under FIML and shown for all participants.

| **Supplementary Table 2.** Differences at Age 70 Between Participants who Provided Data at All Waves (‘Completers’) and Participants who Provided Data at Fewer than 4 Waves (‘Non-completers’) | | | | | | | |
| --- | --- | --- | --- | --- | --- | --- | --- |
|  | Completer | |  | Non-completer | | |  |
| Variable | N |  |  | N |  |  | *p* |
| Walking time | 537 | 3.68 (1.02) |  | 548 | 4.03 (1.26) |  | <.001 |
| FEV_1_ | 539 | 2.47 (.68) |  | 546 | 2.25 (.67) |  | <.001 |
| Grip strength | 538 | 30.30 (10.17) |  | 548 | 28.95 (10.13) |  | .028 |
| Matrix reasoning | 538 | 14.46 (5.02) |  | 548 | 12.55 (5.07) |  | <.001 |
| Block design | 537 | 35.67 (10.13) |  | 548 | 31.94 (10.19) |  | <.001 |
| Spatial span | 536 | 15.09 (2.75) |  | 548 | 14.36 (2.87) |  | <.001 |
| Verbal paired associates | 526 | 27.88 (8.53) |  | 524 | 25.00 (9.49) |  | <.001 |
| Logical memory | 539 | 74.43 (16.87) |  | 548 | 68.54 (18.54) |  | <.001 |
| Digit span backward | 539 | 8.03 (2.37) |  | 551 | 7.44 (2.12) |  | <.001 |
| Digit-symbol | 536 | 59.00 (12.40) |  | 550 | 54.26 (13.02) |  | <.001 |
| Symbol search | 538 | 25.64 (6.58) |  | 548 | 23.79 (6.06) |  | <.001 |
| Reaction time | 538 | .63 (.08) |  | 546 | .66 (.09) |  | <.001 |
| Inspection time | 524 | 113.43 (10.52) |  | 517 | 110.83 (11.33) |  | <.001 |
| Age 11 IQ | 508 | 102.17 (15.15) |  | 520 | 97.88 (14.54) |  | <.001 |
| Age | 539 | 69.50 (.83) |  | 552 | 69.57 (.83) |  | .177 |
| Height (in cm) | 539 | 167.08 (8.89) |  | 551 | 165.77 (8.93) |  | .015 |
| Women | 539 | 267 (49.5) |  | 552 | 276 (50.0) |  | .878 |
| Diabetes | 539 | 35 (6.5) |  | 552 | 56 (10.1) |  | .029 |
| CVD | 539 | 123 (22.8) |  | 552 | 145 (26.3) |  | .186 |
| Stroke | 539 | 18 (3.3) |  | 552 | 36 (6.5) |  | .015 |
| Hypertension | 539 | 193 (35.8) |  | 552 | 240 (43.5) |  | .010 |
| *Note.* Data are shown as mean (SD) or N (%). | | | | | | | |

*Correlations between Individual Cognitive and Physical Tests*

Supplementary Tables 3 and 4 show cross-sectional correlations between cognitive and physical test scores at each wave of the study. At each age, a longer walking time was significantly associated with poorer performance on each of the cognitive tests (mean correlation: *r =* -.202; range: *r =* -.349, *r =* -.062). A stronger grip was associated with better performance on 8 cognitive tests at age 70, 7 cognitive tests at age 73, 6 cognitive tests at age 76, and the same 6 cognitive tests at age 79 (mean correlation across all ages: *r =* .105; range: *r =* -.065, *r =* .262). Higher FEV_1_ was significantly associated with better performance on 8 cognitive ability tests at age 70, 7 cognitive tests at age 73, the same 7 cognitive tests at age 76, and 5 cognitive tests at age 79 (mean correlation across all ages: *r =* .109; range: *r =* -.085, *r =* .258). Overall, we observed weaker and often non-significant associations between the physical measures and tests of verbal memory (verbal paired associates, logical memory, and digit span backwards). Correlations between physical tests across waves, and cognitive tests across waves (grouped according to domain of cognitive function) are shown in Supplementary Tables 5 through 8. Performance on the same cognitive test was strongly correlated across waves (between *r* = 0.854 and *r* = 0.514) as was performance on the same physical tests (between *r* = 0.923 and *r* = 0.507).

| **Supplementary Table 3.** Correlation Matrix for Cognitive and Physical Tests at Ages 70 and 73 | | | | | | | | | | | | |
| --- | --- | --- | --- | --- | --- | --- | --- | --- | --- | --- | --- | --- |
| Variable | 1. | 2. | 3. | 4. | 5. | 6. | 7. | 8. | 9. | 10. | 11. | 12. |
| Age 70 |  |  |  |  |  |  |  |  |  |  |  |  |
| 1. Timed walk | - |  |  |  |  |  |  |  |  |  |  |  |
| 2. FEV1 | -.332** | - |  |  |  |  |  |  |  |  |  |  |
| 3. Grip strength | -.315** | .637** | - |  |  |  |  |  |  |  |  |  |
| 4. Matrix reasoning | -.171** | .198** | .188** | - |  |  |  |  |  |  |  |  |
| 5. Block design | -.214** | .258** | .262** | .571** | - |  |  |  |  |  |  |  |
| 6. Spatial span | -.149** | .212** | .213** | .379** | .401** | - |  |  |  |  |  |  |
| 7. Verbal paired associates | -.064* | -.030 | -.065* | .306** | .272** | .164** | - |  |  |  |  |  |
| 8. Logical memory | -.062* | -.005 | -.047 | .327** | .272** | .236** | .477** | - |  |  |  |  |
| 9. Digit span backward | -.096** | .107** | .090** | .401** | .337** | .324** | .265** | .301** | - |  |  |  |
| 10. Digit-symbol | -.212** | .119** | .000 | .366** | .394** | .307** | .244** | .308** | .302** | - |  |  |
| 11. Symbol search | -.183** | .163** | .108** | .450** | .483** | .409** | .219** | .327** | .344** | .618** | - |  |
| 12. Reaction time | .196** | -.143** | -.097** | -.267** | -.318** | -.362** | -.167** | -.231** | -.253** | -.514** | -.480** | - |
| 13. Inspection time | -.140** | .195** | .216** | .215** | .272** | .269** | .092** | .072* | .174** | .301** | .320** | -.359** |
| Age 73 | 1. | 2. | 3. | 4. | 5. | 6. | 7. | 8. | 9. | 10. | 11. | 12. |
| 1. Timed walk | - |  |  |  |  |  |  |  |  |  |  |  |
| 2. FEV1 | -.312** | - |  |  |  |  |  |  |  |  |  |  |
| 3. Grip strength | -.315** | .613** | - |  |  |  |  |  |  |  |  |  |
| 4. Matrix reasoning | -.226** | .158** | .184** | - |  |  |  |  |  |  |  |  |
| 5. Block design | -.217** | .226** | .247** | .534** | - |  |  |  |  |  |  |  |
| 6. Spatial span | -.208** | .223** | .214** | .358** | .451** | - |  |  |  |  |  |  |
| 7. Verbal paired associates | -.127** | -.037 | -.053 | .285** | .244** | .176** | - |  |  |  |  |  |
| 8. Logical memory | -.114** | .005 | .010 | .327** | .280** | .233** | .521** | - |  |  |  |  |
| 9. Digit span backward | -.147** | .025 | .071* | .347** | .290** | .307** | .267** | .311** | - |  |  |  |
| 10. Digit-symbol | -.308** | .092** | -.011 | .377** | .399** | .323** | .259** | .333** | .331** | - |  |  |
| 11. Symbol search | -.248** | .167** | .123** | .379** | .482** | .392** | .214** | .296** | .326** | .628** | - |  |
| 12. Reaction time | .273** | -.155** | -.107** | -.251** | -.315** | -.350** | -.220** | -.272** | -.225** | -.551** | -.502** | - |
| 13. Inspection time | -.260** | .192** | .186** | .262** | .296** | .285** | .189** | .196** | .194** | .376** | .361** | -.382** |
| *Note.* Correlations highlighted in grey are between tests of physical and cognitive function. Ns for correlation tests at age 70 range between 1,011 and 1,090. Ns for correlation tests at age 73 range between 819 and 866. | | | | | | | | | | | | |

| **Supplementary Table 4.** Correlation Matrix for Cognitive and Physical Tests at Ages 76 and 79 | | | | | | | | | | | | |
| --- | --- | --- | --- | --- | --- | --- | --- | --- | --- | --- | --- | --- |
|  | 1. | 2. | 3. | 4. | 5. | 6. | 7. | 8. | 9. | 10. | 11. | 12. |
| Age 76 |  |  |  |  |  |  |  |  |  |  |  |  |
| 1. Timed walk |  |  |  |  |  |  |  |  |  |  |  |  |
| 2. FEV_1_ | -.258** |  |  |  |  |  |  |  |  |  |  |  |
| 3. Grip strength | -.265** | .586** |  |  |  |  |  |  |  |  |  |  |
| 4. Matrix reasoning | -.200** | .156** | .171** |  |  |  |  |  |  |  |  |  |
| 5. Block design | -.211** | .222** | .229** | .566** |  |  |  |  |  |  |  |  |
| 6. Spatial span | -.148** | .175** | .201** | .414** | .444** |  |  |  |  |  |  |  |
| 7. Verbal paired associates | -.082* | -.059 | -.033 | .305** | .190** | .172** |  |  |  |  |  |  |
| 8. Logical memory | -.119** | -.004 | .042 | .319** | .296** | .253** | .530** |  |  |  |  |  |
| 9. Digit span backward | -.170** | -.031 | .046 | .380** | .318** | .313** | .313** | .322** |  |  |  |  |
| 10. Digit-symbol | -.290** | .100** | .046 | .397** | .436** | .321** | .285** | .403** | .335** |  |  |  |
| 11. Symbol search | -.281** | .157** | .123** | .441** | .532** | .401** | .256** | .358** | .354** | .655** |  |  |
| 12. Reaction time | .271** | -.108** | -.119** | -.285** | -.360** | -.348** | -.248** | -.291** | -.261** | -.556** | -.525** |  |
| 13. Inspection time | -.223** | .219** | .174** | .287** | .345** | .262** | .178** | .185** | .180** | .367** | .406** | -.358** |
| Age 79 |  |  |  |  |  |  |  |  |  |  |  |  |
| 1. Timed walk | - |  |  |  |  |  |  |  |  |  |  |  |
| 2. FEV_1_ | -.334^**^ | - |  |  |  |  |  |  |  |  |  |  |
| 3. Grip strength | -.305^**^ | .640^**^ | - |  |  |  |  |  |  |  |  |  |
| 4. Matrix reasoning | -.264^**^ | .133^**^ | .182^**^ | - |  |  |  |  |  |  |  |  |
| 5. Block design | -.239^**^ | .182^**^ | .243^**^ | .555^**^ | - |  |  |  |  |  |  |  |
| 6. Spatial span | -.179^**^ | .155^**^ | .215^**^ | .402^**^ | .409^**^ | - |  |  |  |  |  |  |
| 7. Verbal paired associates | -.188^**^ | -.085 | -.060 | .323^**^ | .256^**^ | .118^**^ | - |  |  |  |  |  |
| 8. Logical memory | -.190^**^ | .023 | .006 | .411^**^ | .283^**^ | .267^**^ | .553^**^ | - |  |  |  |  |
| 9. Digit span backward | -.162^**^ | -.019 | -.040 | .377^**^ | .312^**^ | .334^**^ | .292^**^ | .345^**^ | - |  |  |  |
| 10. Digit-symbol | -.344^**^ | .036 | .007 | .382^**^ | .422^**^ | .396^**^ | .318^**^ | .412^**^ | .410^**^ | - |  |  |
| 11. Symbol search | -.263^**^ | .080 | .096^*^ | .461^**^ | .534^**^ | .410^**^ | .264^**^ | .377^**^ | .391^**^ | .649^**^ | - |  |
| 12. Reaction time | .349^**^ | -.085^*^ | -.107^*^ | -.314^**^ | -.345^**^ | -.338^**^ | -.262^**^ | -.314^**^ | -.300^**^ | -.613^**^ | -.468^**^ | - |
| 13. Inspection time | -.295^**^ | .167^**^ | .200^**^ | .351^**^ | .319^**^ | .275^**^ | .199^**^ | .311^**^ | .175^**^ | .420^**^ | .431^**^ | -.365^**^ |
| *Note.* Correlations highlighted in grey are between tests of physical and cognitive function. Ns for correlation tests at age 76 range between 696 and 630. Ns for correlation tests at age 73 range between 548 and 438. | | | | | | | | | | | | |

| **Supplementary Table 5.** Correlation Matrix for Physical Function Measures Taken at Ages 70, 73, 76 and 79 | | | | | | | | | | | |
| --- | --- | --- | --- | --- | --- | --- | --- | --- | --- | --- | --- |
| Variable |  |  |  |  |  |  |  |  |  |  |  |
| 1. Timed walk 70 | - |  |  |  |  |  |  |  |  |  |  |
| 2. Timed walk 73 | .631** | - |  |  |  |  |  |  |  |  |  |
| 3. Timed walk 76 | .507** | .614** | - |  |  |  |  |  |  |  |  |
| 4. Timed walk 79 | .534** | .559** | .690** | - |  |  |  |  |  |  |  |
| 5. FEV1 70 | -.332** | -.319** | -.263** | -.295** | - |  |  |  |  |  |  |
| 6. FEV1 73 | -.276** | -.312** | -.269** | -.303** | .923** | - |  |  |  |  |  |
| 7. FEV1 76 | -.273** | -.300** | -.258** | -.294** | .877** | .876** | - |  |  |  |  |
| 8. FEV1 79 | -.293** | -.314** | -.287** | -.334** | .876** | .855** | .836** | - |  |  |  |
| 9. Grip strength 70 | -.315** | -.278** | -.270** | -.268** | .637** | .602** | .602** | .630** | - |  |  |
| 10. Grip strength 73 | -.296** | -.315** | -.279** | -.258** | .640** | .613** | .618** | .630** | .918** | - |  |
| 11. Grip strength 76 | -.293** | -.308** | -.265** | -.300** | .611** | .590** | .586** | .635** | .845** | .871** | - |
| 12. Grip strength 79 | -.327** | -.294** | -.329** | -.305** | .654** | .636** | .639** | .640** | .881** | .888** | .877** |
| *Note.* Correlations highlighted in grey are between different physical function measures. | | | | | | | | | | | |

| **Supplementary Table 6.** Correlation Matrix for Visuospatial Ability Measures Taken at Ages 70, 73, 76 and 79 | | | | | | | | | | | |
| --- | --- | --- | --- | --- | --- | --- | --- | --- | --- | --- | --- |
| Variable | 1. | 2. | 3. | 4. | 5. | 6. | 7. | 8. | 9. | 10. | 11. |
| 1. Matrix reasoning 70 | - |  |  |  |  |  |  |  |  |  |  |
| 2. Matrix reasoning 73 | .648** | - |  |  |  |  |  |  |  |  |  |
| 3. Matrix reasoning 76 | .631** | .636** | - |  |  |  |  |  |  |  |  |
| 4. Matrix reasoning 79 | .620** | .642** | .641** | - |  |  |  |  |  |  |  |
| 5. Block design 70 | .571** | .546** | .561** | .538** | - |  |  |  |  |  |  |
| 6. Block design 73 | .521** | .534** | .510** | .527** | .759** | - |  |  |  |  |  |
| 7. Block design 76 | .547** | .541** | .566** | .566** | .759** | .756** | - |  |  |  |  |
| 8. Block design 79 | .493** | .494** | .489** | .555** | .720** | .750** | .782** | - |  |  |  |
| 9. Spatial span 70 | .379** | .352** | .371** | .351** | .401** | .400** | .409** | .334** | - |  |  |
| 10. Spatial span 73 | .374** | .358** | .382** | .370** | .416** | .451** | .440** | .392** | .558** | - |  |
| 11. Spatial span 76 | .304** | .341** | .414** | .375** | .360** | .369** | .444** | .312** | .582** | .567** | - |
| 12. Spatial span 79 | .328** | .311** | .384** | .402** | .388** | .383** | .425** | .409** | .567** | .588** | .579** |
| *Note.* Correlations highlighted in grey are between different visuospatial ability measures. | | | | | | | | | | | |

| **Supplementary Table 7.** Correlation Matrix for Verbal Memory Measures Taken at Ages 70, 73, 76 and 79 | | | | | | | | | | | |
| --- | --- | --- | --- | --- | --- | --- | --- | --- | --- | --- | --- |
| Variable |  |  |  |  |  |  |  |  |  |  |  |
| 1. Verbal paired associates 70 | - |  |  |  |  |  |  |  |  |  |  |
| 2. Verbal paired associates 73 | .715** | - |  |  |  |  |  |  |  |  |  |
| 3. Verbal paired associates 76 | .668** | .703** | - |  |  |  |  |  |  |  |  |
| 4. Verbal paired associates 79 | .605** | .643** | .757** | - |  |  |  |  |  |  |  |
| 5. Logical memory 70 | .477** | .379** | .360** | .316** | - |  |  |  |  |  |  |
| 6. Logical memory 73 | .436** | .521** | .451** | .383** | .702** | - |  |  |  |  |  |
| 7. Logical memory 76 | .398** | .396** | .530** | .442** | .609** | .742** | - |  |  |  |  |
| 8. Logical memory 79 | .349** | .368** | .478** | .553** | .566** | .659** | .785** | - |  |  |  |
| 9. Digit span backward 70 | .265** | .249** | .238** | .191** | .301** | .295** | .264** | .226** | - |  |  |
| 10. Digit span backward 73 | .244** | .267** | .278** | .205** | .275** | .311** | .294** | .227** | .638** | - |  |
| 11. Digit span backward 76 | .245** | .257** | .313** | .242** | .275** | .278** | .322** | .266** | .651** | .682** | - |
| 12. Digit span backward 79 | .209** | .245** | .302** | .292** | .254** | .315** | .343** | .345** | .639** | .653** | .682** |
| *Note.* Correlations highlighted in grey are between different verbal memory measures. | | | | | | | | | | | |

| **Supplementary Table 8.** Correlation Matrix for Processing Speed Cognitive Ability Measures Taken at Ages 70, 73, 76 and 79 | | | | | | | | | | | | | | | |
| --- | --- | --- | --- | --- | --- | --- | --- | --- | --- | --- | --- | --- | --- | --- | --- |
| Variable | 1. | 2. | 3. | 4. | 5. | 6. | 7. | 8. | 9. | 10. | 11. | 12. | 13. | 14. | 15. |
| 1. Digit-symbol 70 | - |  |  |  |  |  |  |  |  |  |  |  |  |  |  |
| 2. Digit-symbol 73 | .847^**^ | - |  |  |  |  |  |  |  |  |  |  |  |  |  |
| 3. Digit-symbol 76 | .774^**^ | .832^**^ | - |  |  |  |  |  |  |  |  |  |  |  |  |
| 4. Digit-symbol 79 | .728^**^ | .812^**^ | .854^**^ | - |  |  |  |  |  |  |  |  |  |  |  |
| 5. Symbol search 70 | .618^**^ | .592^**^ | .558^**^ | .556^**^ | - |  |  |  |  |  |  |  |  |  |  |
| 6. Symbol search 73 | .583^**^ | .628^**^ | .580^**^ | .526^**^ | .669^**^ | - |  |  |  |  |  |  |  |  |  |
| 7. Symbol search 76 | .575^**^ | .619^**^ | .655^**^ | .614^**^ | .631^**^ | .671^**^ | - |  |  |  |  |  |  |  |  |
| 8. Symbol search 79 | .503^**^ | .564^**^ | .578^**^ | .649^**^ | .620^**^ | .631^**^ | .701^**^ | -- |  |  |  |  |  |  |  |
| 9. Reaction time 70 | -.514^**^ | -.526^**^ | -.485^**^ | -.462^**^ | -.480^**^ | -.472^**^ | -.445^**^ | -.389^**^ |  |  |  |  |  |  |  |
| 10. Reaction time 73 | -.517^**^ | -.551^**^ | -.532^**^ | -.510^**^ | -.447^**^ | -.502^**^ | -.500^**^ | -.426^**^ | .749^**^ | - |  |  |  |  |  |
| 11. Reaction time 76 | -.436^**^ | -.479^**^ | -.556^**^ | -.531^**^ | -.402^**^ | -.402^**^ | -.525^**^ | -.464^**^ | .674^**^ | .705^**^ | - |  |  |  |  |
| 12. Reaction time 79 | -.447^**^ | -.514^**^ | -.528^**^ | -.613^**^ | -.416^**^ | -.378^**^ | -.473^**^ | -.468^**^ | .614^**^ | .665^**^ | .684^**^ | - |  |  |  |
| 13. Inspection time 70 | .301^**^ | .312^**^ | .296^**^ | .288^**^ | .320^**^ | .278^**^ | .265^**^ | .287^**^ | -.359^**^ | -.357^**^ | -.321^**^ | -.237^**^ | - |  |  |
| 14. Inspection time 73 | .314^**^ | .376^**^ | .323^**^ | .303^**^ | .347^**^ | .361^**^ | .341^**^ | .329^**^ | -.342^**^ | -.382^**^ | -.329^**^ | -.258^**^ | .586^**^ | - |  |
| 15. Inspection time 76 | .285^**^ | .338^**^ | .367^**^ | .328^**^ | .348^**^ | .340^**^ | .406^**^ | .379^**^ | -.284^**^ | -.313^**^ | -.358^**^ | -.291^**^ | .516^**^ | .597^**^ | - |
| 16. Inspection time 79 | .286^**^ | .359^**^ | .363^**^ | .420^**^ | .287^**^ | .242^**^ | .353^**^ | .431^**^ | -.309^**^ | -.327^**^ | -.360^**^ | -.365^**^ | .514^**^ | .571^**^ | .528^**^ |
| *Note.* Correlations highlighted in grey are between different processing speed measures. | | | | | | | | | | | | | | | |

*Within-cognitive and Within-physical Intercept and Slope Correlations*

We observed a moderate to strong correlation between slopes of cognitive functions (between *r =* 0.685 and *r* = 0.878), and a moderate correlation between slopes of FEV_1_ and grip strength (*r* = 0.577) but not between slopes of FEV_1_ and walking speed or between slopes of walking speed and grip strength (see Supplementary Table 9).

| **Supplementary Table 9.** Within-cognitive and Within-physical Intercept and Slope Correlations | | | | | |
| --- | --- | --- | --- | --- | --- |
|  | | Intercepts | | Slopes | |
|  | | *r* | *p* | *r* | *p* |
| **Cognitive** | **Cognitive** |  |  |  |  |
| Speed | Memory | 0.597 | <.001 | 0.685 | <.001 |
| Spatial | Memory | 0.737 | <.001 | 0.760 | <.001 |
| Speed | Spatial | 0.759 | <.001 | 0.878 | <.001 |
| **Physical** | **Physical** |  |  |  |  |
| Grip strength | Walking time | -0.296 | <.001 | -0.168 | 0.093 |
| FEV_1_ | Walking time | -0.290 | <.001 | -0.111 | 0.361 |
| Grip strength | FEV_1_ | 0.281 | <.001 | 0.577 | 0.002 |
| *Note.* Estimates from trivariate growth curve models of cognitive or physical functions. Cognitive measures were adjusted for age at time of testing and sex. Physical measures were adjusted for age and height at time of testing and for sex. | | | | | |

*Correlations Between Cognitive and Physical Function Intercepts and Slopes*

Supplementary Table 10 shows correlations between intercepts of cognitive function and slopes of physical function, and between intercepts of physical function and slopes of cognitive function. In the fully adjusted model, only correlations between processing speed intercept and walking time slope, and walking time intercept and processing speed slope were statistically significant. Shorter walking time at age 70 was associated with less decline in processing speed (*r* = - 0.202), and faster processing speed at age 70 was associated with less increase in walking time (*r* = -0.206).

| **Supplementary Table 10.** Correlations between Intercepts and Slopes of Physical and Cognitive Function | | | | | | | | | |
| --- | --- | --- | --- | --- | --- | --- | --- | --- | --- |
|  | | Cognitive intercept  Physical slope | | | | Physical intercept  cognitive slope | | | |
| Variable | | Minimally  adjusted | | Fully  adjusted | | Minimally  adjusted | | Fully  adjusted | |
| Physical | Cognitive | *r* | *p* | *r* | *p* | *r* | *p* | *r* | *p* |
| Walking | Memory | -0.069 | 0.266 | -0.023 | 0.750 | -0.106 | 0.119 | -0.070 | 0.324 |
| Walking | Speed | **-0.285** | <.001 | **-0.202** | <.001 | **-0.233** | 0.001 | **-0.206** | 0.005 |
| Walking | Spatial | **-0.191** | <.001 | -0.123 | 0.042 | -0.067 | 0.588 | -0.126 | 0.340 |
| Grip | Memory | -0.017 | 0.862 | 0.081 | 0.450 | 0.100 | 0.061 | 0.043 | 0.435 |
| Grip | Speed | -0.073 | 0.427 | -0.016 | 0.861 | **0.151** | 0.005 | 0.107 | 0.053 |
| Grip | Spatial | 0.040 | 0.655 | 0.160 | 0.089 | -0.047 | 0.608 | -0.030 | 0.763 |
| FEV_1_ | Memory | -0.390 | 0.047 | -0.149 | 0.424 | 0.061 | 0.239 | 0.002 | 0.963 |
| FEV_1_ | Speed | -0.270 | 0.103 | 0.058 | 0.698 | 0.087 | 0.093 | 0.038 | 0.475 |
| FEV_1_ | Spatial | -0.234 | 0.154 | 0.115 | 0.480 | 0.108 | 0.233 | 0.157 | 0.098 |
| *Note.* Estimates from model 0 (with no coupling effects and no auto-proportional effects). Estimates in bold are statistically significant. Minimally adjusted estimates are adjusted for sex and age at time of testing; fully adjusted estimates are additionally adjusted for age 11 IQ, height at time of testing, and history of chronic disease (diabetes, stroke, CVD, and hypertension). Memory = verbal memory; speed = processing speed; spatial = visuospatial ability. | | | | | | | | | |

*Correlation between Concurrent Change Scores.*

An assumption of model 0 (no coupling effects and no auto-proportional effects) is that cognitive and physical functions follow a linear pattern of change. However, it is possible that changes in these variables are non-linear. Therefore, in addition to examining correlations between concurrent linear changes (estimated in model 0) we report correlations between concurrent change scores estimated in model 1 (which additionally included auto-proportional effects from the variable’s earlier level and change to its own upcoming change). The additional paths in model 1 transform static linear changes in physical and cognitive function to nonlinear exponential changes (7). Model estimated correlations between concurrent change scores are shown in Supplementary Table 11. Estimated correlations between changes in walking time and concurrent changes in each domain of cognitive ability were negative (an increase in walking time was related to a decrease in cognitive ability), statistically significant, and similar in magnitude across time. Estimated correlations between changes in grip strength and concurrent changes in visuospatial ability were not statistically significant. Correlations between concurrent changes in grip strength and verbal memory or processing speed were positive and similar in magnitude across the first two intervals (ages 70-73 and ages 73-76). However, correlations between changes occurring over the final interval (between ages 76-79) were negative.

| **Supplementary Table 11.** Model 1 Estimated Correlations Between Concurrent Changes in Physical and Cognitive Functions. | | | | | | | |
| --- | --- | --- | --- | --- | --- | --- | --- |
| Model | Changes  ages  70-73 | | *p* | Changes ages  73-76 | *p* | Changes ages  76-79 | *p* |
| Memory and walking | -0.213 | <.001 | | -0.305 | <.001 | -0.293 | <.001 |
| Speed and walking | -0.539 | <.001 | | -0.522 | <.001 | -0.500 | <.001 |
| Spatial and walking | -0.350 | <.001 | | -0.478 | <.001 | -0.362 | <.001 |
| Memory and grip strength | 0.208 | .005 | | 0.258 | .001 | -0.223 | .002 |
| Speed and grip strength | 0.241 | .001 | | 0.270 | <.001 | -0.238 | .001 |
| Spatial and grip strength | 0.230 | .079 | | 0.224 | .089 | -0.085 | .508 |

| **Supplementary Table 12.** Changes in Fit of the Bivariate Latent Change Score Models | | | | | | | | |
| --- | --- | --- | --- | --- | --- | --- | --- | --- |
| Model | Parameters | X^2^ | df | CFI | BIC | RMSEA | ^△^X^2^ | *p* |
| Verbal memory and walking time | | | | | | | | |
| 1. No coupling | 82 | 444 | 170 | .962 | 44,533 | .038 |  |  |
| **2. △ walking 🡪 △ memory** | **84** | **427** | **168** | **.964** | **44,529** | **.038** | **-17** | **<.001** |
| 3. △ memory 🡪 walking | 84 | 444 | 168 | .961 | 44,547 | .039 | - | - |
| Processing speed and walking time | |  |  |  |  |  |  |  |
| **1. No coupling** | **91** | **761** | **259** | **.952** | **50,832** | **.042** |  |  |
| 2. △ walking 🡪 △ processing | 93 | 760 | 257 | .952 | 50,845 | .042 | -1 | .607 |
| 3. △ processing 🡪 △ walking | 93 | 759 | 257 | .952 | 50,845 | .042 | -2 | .368 |
| Visuospatial ability and walking time | |  |  |  |  |  |  |  |
| 1. No coupling | 82 | 258 | 170 | .987 | 43,836 | .022 |  |  |
| 2. △ walking 🡪 △ visuospatial^a^ | 83 | 255 | 169 | .988 | 39,445 | .022 | -3 | 0.083 |
| **3. △ visuospatial 🡪 △ walking** | **84** | **238** | **168** | **.990** | **39,435** | **.020** | **-20** | **<.001** |
| Verbal memory and grip strength | |  |  |  |  |  |  |  |
| 1. No coupling | 82 | 472 | 170 | .971 | 53,480 | .040 |  |  |
| **2. △ grip 🡪 △ memory** | **84** | **446** | **168** | **.974** | **53,469** | **.039** | **-26** | **<.001** |
| 3. △ memory 🡪 △ grip | 84 | 462 | 168 | .972 | 53,485 | .040 | -10 | .007 |
| 4. Full coupling^b^ | 86 | 440 | 166 | .974 | 53,477 | .039 | -32 | .050 |
| Processing speed and grip strength |  |  |  |  |  |  |  |  |
| 1. No coupling | 91 | 759 | 259 | .964 | 59,889 | .042 |  |  |
| 2. △ grip 🡪 △ processing | 93 | 756 | 257 | .964 | 59,901 | .042 | -3 | .223 |
| **3. △ processing 🡪 △ grip** | **93** | **739** | **257** | **.965** | **59,884** | **.041** | **-20** | **<.001** |
| Visuospatial ability and grip strength |  |  |  |  |  |  |  |  |
| 1. No coupling | 82 | 279 | 170 | .990 | 52,797 | .024 |  |  |
| 2. △ walking 🡪 △ visuospatial | 84 | 276 | 168 | .990 | 52,808 | .024 | -3 | .223 |
| **3. △ visuospatial 🡪 △ grip** | **84** | **243** | **168** | **.993** | **52,774** | **.020** | **-36** | **<.001** |
| *Note.* Models shown in bold are the best fitting models. The full coupling model, model 4, was only run if both models 2 and 3 resulted in improved fit over model 1. ^a^ To allow this model to converge, the path from visuospatial ability level at age 73 to visuospatial ability change (between ages 73 and 76) was not estimated. ^b^ Full coupling model compared to model 2 (with paths from change in grip strength to upcoming change in memory). | | | | | | | | |

| **Supplementary Table 13**. Parameter Estimates from Model 2 of Verbal Memory and Walking Time | | | | |
| --- | --- | --- | --- | --- |
| Path | *β* | B | B 99% CI | *p* |
| Memory 70 🡪△ Memory 70-73 | 0.481 | 0.095 | -0.097, 0.287 | .203 |
| Memory 73 🡪△ Memory 73-76 | -1.632 | -0.714 | -1.886, 0.458 | .117 |
| Memory 76 🡪△ Memory 76-79 | -0.285 | -0.073 | -0.253, 0.107 | .295 |
| △ Memory 70-73 🡪△ Memory 73-76 | 1.641 | 4.171 | -2.944,11.286 | .131 |
| △ Memory 73-76 🡪△ Memory 76-79 | 0.547 | 0.351 | -0.036, 0.738 | .019 |
| Walking 70 🡪△ Walking 70-73 | 0.065 | 0.025 | -0.626, 0.676 | .920 |
| Walking 73 🡪△ Walking 73-76 | -0.270 | -0.120 | -0.749, 0.510 | .624 |
| Walking 76 🡪△ Walking 76-79 | -0.045 | -0.018 | -0.617, 0.580 | .937 |
| △ Walking 70-73 🡪△ Walking 73-76 | 0.586 | 0.894 | -0.580, 2.368 | .118 |
| △Walking 73-76 🡪△ Walking 76-79 | 0.548 | 0.694 | -0.381, 1.769 | .096 |
| △ Walking 70-73 🡪△ Memory 73-76 | -0.344 | -0.334 | -0.766, 0.097 | .046 |
| **△ Walking 73-76 🡪△ Memory 76-79** | **-0.611** | **-0.250** | **-0.405, -0.094** | **<.001** |

| **Supplementary Table 14.** Parameter Estimates from Model 1 of Processing Speed and Walking Time | | | | |
| --- | --- | --- | --- | --- |
| Path | *β* | B | B 99% CI | *p* |
| Speed 70 🡪△ Speed 70-73 | -0.183 | -0.023 | -0.181, 0.136 | .712 |
| Speed 73 🡪 △ Speed 73-76 | -0.389 | -0.105 | -0.301, 0.090 | .164 |
| Speed 76 🡪 △ Speed 76-79 | -0.289 | -0.086 | -0.261, 0.090 | .208 |
| **△ Speed 70-73🡪△ Speed 73-76** | **0.709** | **1.633** | **0.757, 2.510** | **<.001** |
| **△ Speed 73-76 🡪△ Speed 76-79** | **0.790** | **0.971** | **0.642, 1.301** | **<.001** |
| Walking 70 🡪△ Walking 70-73 | -0.128 | -0.048 | -0.643, 0.547 | .835 |
| Walking 73 🡪△ Walking 73-76 | -0.506 | -0.248 | -0.822, 0.325 | .264 |
| Walking 76 🡪△ Walking 76-79 | -0.178 | -0.071 | -0.608, 0.465 | .731 |
| △ Walking 70-73 🡪△ Walking 73-76 | 0.768 | 1.335 | -0.388, 3.058 | .046 |
| △ Walking 73-76 🡪△ Walking 76-79 | 0.620 | 0.709 | -0.183, 1.601 | .041 |

| **Supplementary Table 15.** Parameter Estimates from Model 3 of Visuospatial Ability and Walking Time | | | | |
| --- | --- | --- | --- | --- |
| Path | *β* | B | B 99% CI | *p* |
| Vis 70 🡪 △ Visuospatial 70-73 | 0.087 | 0.004 | -0.082,0.089 | 0.908 |
| Visuospatial 73 🡪 △ Visuospatial 73-76 | 0.708 | 0.053 | -0.027, 0.134 | 0.086 |
| Visuospatial 76 🡪 △ Visuospatial 76-79 | -0.246 | -0.018 | -0.120, 0.084 | 0.645 |
| △ Visuospatial 70-73 🡪 △ Visuospatial 73-76 | 0.386 | 0.641 | -0.468, 1.750 | 0.137 |
| △ Visuospatial 73-76 🡪 △ Visuospatial 76-79 | 0.410 | 0.412 | -0.074, 0.898 | 0.029 |
| Walk 70 🡪△ Walking 70-73 | 0.050 | 0.019 | -0.864, 0.903 | 0.955 |
| Walking 73 🡪 △ Walking 73-76 | -0.307 | -0.188 | -1.079, 0.703 | 0.587 |
| Walking 76 🡪 △ Walking 76-79 | -1.496 | -0.839 | -1.917, 0.239 | 0.045 |
| △ Walking 70-73 🡪 △ Walking 73-76 | 0.763 | 1.558 | -0.243, 3.358 | 0.026 |
| △ Walking 73-76 🡪 △ Walking 76-79 | 0.222 | 0.295 | -1.381, 1.971 | 0.650 |
| △ Visuospatial 70-73 🡪 △ Walking 73-76 | 0.085 | 2.036 | -14.214, 18.288 | 0.747 |
| △ Visuospatial 73-76 🡪 △ Walking 76-79 | -1.582 | -30.328 | -62.462, 1.806 | 0.015 |

| **Supplementary Table 16.** Parameter Estimates from Model 2 of Verbal Memory and Grip Strength | | | | |
| --- | --- | --- | --- | --- |
| Path | *β* | B | B 99% CI | *p* |
| Memory 70 🡪 △ Memory 70-73 | -0.815 | -0.233 | -0.985, 0.519 | .425 |
| **Memory 73 🡪 △ Memory 73-76** | **-1.153** | **-0.457** | **-0.915, 0.000** | **.010** |
| Memory 76 🡪△ Memory 76-79 | -1.202 | -0.358 | -0.985, 0.269 | .141 |
| △ Memory 70-73 🡪△ Memory 73-76. | 0.714 | 1.086 | -1.075, 3.247 | .196 |
| △ Memory 73-76 🡪△ Memory 76-79 | 0.569 | 0.478 | -0.448, 1.404 | .183 |
| Grip 70 🡪△ Grip 70-73 | 1.995 | 0.136 | -0.168, 0.441 | .249 |
| Grip 73 🡪△ Grip 73-76 | 0.647 | 0.174 | -0.132, 0.481 | .143 |
| Grip 76 🡪△ Grip 76-79 | 0.701 | 0.082 | -0.228, 0.391 | .496 |
| △ Grip 70-73 🡪△ Grip 73-76 | 0.733 | 2.835 | -1.140, 6.810 | .066 |
| **△ Grip 73-76 🡪△ Grip 76-79** | **-1.742** | **-0.755** | **-1.330, -0.180** | **.001** |
| **△ Grip 70-73 🡪△ Memory 73-76** | **0.662** | **0.304** | **0.059, 0.548** | **.001** |
| **△ Grip 73-76 🡪△ Memory 76-79** | **0.976** | **0.097** | **0.030, 0.165** | **<.001** |

| **Supplementary Table 17.** Parameter Estimates from Model 3 of Processing Speed and Grip Strength | | | | |
| --- | --- | --- | --- | --- |
| Path | *β* | B | B 99% CI | *p* |
| Speed 70 🡪△ Speed 70-73 | 0.088 | 0.011 | -0.148, 0.170 | 0.859 |
| Speed 73 🡪 △ Speed 73-76 | -0.396 | -0.105 | -0.293, 0.084 | 0.153 |
| Speed 76 🡪△ Speed 76-79 | -0.171 | -0.049 | -0.221, 0.122 | 0.459 |
| **△ Speed 70-73 🡪△ Speed 73-76** | **0.747** | **1.686** | **0.772, 2.600** | **<.001** |
| **△ Speed 73-76 🡪△ Speed 76-79** | **0.743** | **0.898** | **0.575, 1.222** | **<.001** |
| Grip 70 🡪△ Grip 70-73 | -1.063 | -0.101 | -0.514, 0.312 | 0.528 |
| Grip 73 🡪△ Grip 73-76 | -0.090 | -0.028 | -0.459, 0.403 | 0.868 |
| Grip 76 🡪△ Grip 76-79 | -0.624 | -0.119 | -0.542, 0.303 | 0.467 |
| **△ Grip 70-73 🡪△ Grip 73-76** | **0.739** | **2.333** | **-0.005, 4.671** | **0.010** |
| **△ Grip 73-76 🡪△ Grip 76-79** | **-1.253** | **-0.790** | **-1.403, -0.177** | **0.001** |
| △ Speed 70-73 🡪△ Grip 73-76 | 0.359 | 15.433 | -7.046, 37.912 | 0.077 |
| **△ Speed 73-76 🡪△ Grip 76-79** | **0.642** | **7.703** | **2.638, 12.767** | **<.001** |

| **Supplementary Table 18.** Parameter Estimates from Model 3 of Visuospatial Ability and Grip Strength | | | | |
| --- | --- | --- | --- | --- |
| Path | *β* | B | B 99% CI | *p* |
| Visuospatial 70 🡪 △ Visuospatial 70-73 | 0.711 | 0.043 | -0.187, 0.274 | 0.627 |
| Visuospatial 73 🡪 △ Visuospatial 73-76 | 0.180 | 0.022 | -0.215, 0.258 | 0.814 |
| Visuospatial 76 🡪 △ Visuospatial 76-79 | -0.073 | -0.008 | -0.248, 0.232 | 0.929 |
| △Visuospatial 70-73 🡪 △ Visuospatial 73-76 | 0.496 | 0.978 | -0.590, 2.546 | 0.108 |
| △Visuospatial 73-76 🡪 △ Visuospatial 76-79 | 0.408 | 0.385 | -0.178, 0.948 | 0.078 |
| Grip 70 🡪 △ Grip 70-73 | -1.889 | -0.220 | -0.974, 0.534 | 0.452 |
| Grip 73 🡪△ Grip 73-76 | -0.203 | -0.067 | -0.807, 0.673 | 0.816 |
| Grip 76 🡪 △ Grip 76-79 | -0.600 | -0.157 | -0.938, 0.625 | 0.606 |
| **△Grip 70-73 🡪 △ Grip 73-76** | **0.895** | **2.415** | **0.182, 4.647** | **0.005** |
| △Grip 73-76 🡪 △Grip 76-79 | -0.951 | -0.781 | -1.871, 0.308 | 0.065 |
| △Visuospatial 70-73 🡪 △ Grip 73-76 | 0.730 | 53.536 | -10.183, 117.254 | 0.030 |
| **△Visuospatial 73-76 🡪△ Grip 76-79** | **0.965** | **29.452** | **8.868, 50.037** | **<.001** |

| **Supplementary Table 19.** Associations Between Time-invariant Covariate Variables and Physical and Cognitive Function Levels and Slopes | | | | | |
| --- | --- | --- | --- | --- | --- |
|  | Predictors | | | | |
|  |  | Age 11 IQ |  | Sex = female |  |
| Outcome | Variable | B | *p* | B | *p* |
| Walking time | Intercept | -0.111 | <.001 | 0.366 | <.001 |
|  | Slope | 0.009 | .223 | -0.056 | .046 |
| Grip strength | Intercept | 0.234 | .056 | -12.344 | <.001 |
|  | Slope | -0.051 | 0.137 | 0.822 | .248 |
| Verbal memory | Intercept | 0.261 | <.001 | 0.188 | .002 |
|  | Slope | 0.043 | .304 | 0.115 | .009 |
| Processing speed | Intercept | 0.173 | <.001 | 0.180 | <.001 |
|  | Slope | 0.033 | .007 | 0.073 | .001 |
| Visuospatial ability | Intercept | 0.268 | <.001 | -0.194 | .001 |
|  | Slope | 0.061 | .213 | -0.019 | .494 |
| *Note.* Estimates are unstandardized. Each physical and cognitive function modelled separately. Auto-proportional effects are included. All covariate variables including time varying covariates entered simultaneously in each physical or cognitive function model. | | | | | |

| **Supplementary Table 20.** Associations between Time-varying Covariate Variables and Physical and Cognitive Functions at Ages 70 and 79 | | | | | | | | | | | | | |
| --- | --- | --- | --- | --- | --- | --- | --- | --- | --- | --- | --- | --- | --- |
|  | Predictors | | | | | | | | | | | | |
|  |  | Age |  | CVD |  | Stroke |  | Diabetes |  | hypertension | | Height |  |
| Outcome | Wave | Estimate | *p* | Estimate | *p* | Estimate | *p* | Estimate | *p* | Estimate | *p* | Estimate | *p* |
| Walking time | 1 | 0.013 | .220 | 0.199 | .005 | 0.121 | .398 | 0.352 | .001 | 0.149 | .014 | -0.098 | .069 |
|  | 4 | -0.016 | .637 | 0.018 | .878 | -0.308 | .110 | 0.074 | .673 | 0.087 | .464 | -0.074 | .241 |
| Grip strength | 1 | -0.198 | .001 | -0.628 | .126 | -1.671 | .037 | -1.969 | .002 | -0.148 | .684 | 2.962 | <.001 |
|  | 4 | 0.212 | .066 | 0.036 | .920 | 0.346 | .509 | 0.310 | .560 | 0.104 | .768 | 0.212 | .615 |
| Verbal memory | 1 | -0.042 | <.001 | 0.085 | .080 | 0.135 | .232 | 0.054 | .513 | -0.044 | .315 | 0.047 | .160 |
|  | 4 | -0.049 | <.001 | 0.023 | .612 | -0.058 | .365 | -0.006 | .923 | 0.033 | .440 | 0.062 | .134 |
| Processing speed | 1 | -0.025 | <.001 | -0.089 | .009 | -0.256 | <.001 | -0.063 | .237 | -0.023 | .408 | 0.137 | <.001 |
|  | 4 | -0.006 | .398 | 0.016 | .574 | -0.053 | .222 | -0.001 | .976 | 0.049 | .078 | 0.082 | .014 |
| Visuospatial ability | 1 | -0.028 | <.001 | -0.027 | .536 | -0.193 | .018 | -0.083 | .249 | -0.025 | .502 | 0.132 | <.001 |
|  | 4 | -0.034 | .001 | -0.012 | .764 | 0.000 | .994 | -0.075 | .208 | 0.028 | .420 | 0.069 | .352 |
| *Note.* Estimates are unstandardized. Associations with each physical and cognitive function modelled separately. Auto-proportional effects are included. All covariate variables, including time-invariant covariates entered simultaneously in each physical or cognitive function model. Associations shown at waves 1 and 4 only but estimated at each wave of the study. | | | | | | | | | | | | | |

*Changes to Fully-adjusted Models*

The initial fully-adjusted model of grip strength and verbal memory did not converge on within bounds estimates: the residual variance of the slope for grip strength was estimated as negative (this error can occur when a construct has low variance due to overcorrection). This error was remedied by excluding hypertension from the model (this covariate variable was not significantly associated verbal memory or grip strength at any wave of the study). The initial fully-adjusted model of grip strength and visuospatial ability did not converge (this problem can occur if variables are on very different scales). Dividing grip strength scores at each wave by 10 (to reduce the variance of these variables) and excluding hypertension from the analysis (as residual variance of the slopes were also estimated as negative) resulted in an acceptable model. Hypertension was not significantly related to grip strength or visuospatial ability at any wave of the study.

*Subsidiary Analysis: Lead-lag Coupling Effects between Levels and Changes*

Firstly, we identified the best fitting level to change model by comparing four different options 1) a baseline model with only auto-proportional effects (from the variable’s level to its own upcoming change); 2) a model with additional paths from earlier levels of physical function to upcoming changes in cognitive function; 3) a model with additional paths from earlier levels of cognitive function to upcoming changes in physical function; 4) a model with bi-directional coupling effects (combining the paths from models 2 and 3). The best fitting models of walking time and verbal memory, walking time and processing speed, grip strength and verbal memory, and grip strength and processing speed included unidirectional paths from earlier levels of physical function to upcoming changes in cognitive function. However, none of these path estimates were close to statistically significant (all *p*s > 0.04) suggesting that the analysis was underpowered to detect individual coupling effects. Models of walking time or grip strength and visuospatial ability were not improved by inclusion of any lead-lag coupling effects. See Supplementary Table 21 for results of these model comparisons. We then re-ran the main analysis (comparison of models 1-4), for the relevant combinations of physical and cognitive functions (verbal memory and walking time, processing speed and walking time, verbal memory and grip strength, and processing speed and grip strength) additionally including physical level to cognitive change paths in each model (models 1-4). Supplementary Table 22 shows the results of these comparisons.

| **Supplementary Table 21.** Changes in Fit of the Bivariate Latent Change Score Models with Coupling Effects from Earlier Levels to Upcoming Changes | | | | | | | | |
| --- | --- | --- | --- | --- | --- | --- | --- | --- |
| Model | Parameters | X^2^ | df | CFI | BIC | RMSEA | △  X^2^ | *p* |
| Verbal memory and walking time | | | | | | | | |
| 1. No coupling | 78 | 481 | 174 | .957 | 44,542 | .040 |  |  |
| **2. walking 🡪 △ memory** | **81** | **426** | **171** | **.964** | **44,508** | **.037** | **55** | **<.001** |
| 3. memory 🡪 walking | 81 | 479 | 171 | .957 | 44,560 | .041 | 2 | .572 |
| Processing speed and walking time | |  |  |  |  |  |  |  |
| 1. No coupling | 87 | 809 | 263 | .948 | 50,853 | .044 |  |  |
| **2. walking 🡪 △ processing** | **90** | **772** | **260** | **.951** | **50,836** | **.042** | **37** | **<.001** |
| 3. processing 🡪 △ walking | 90 | 801 | 260 | .948 | 50,866 | .044 | 8 | .046 |
| Visuospatial ability and walking time | |  |  |  |  |  |  |  |
| **1. No coupling** | **78** | **269** | **174** | **.986** | **43,826** | **.022** |  |  |
| 2. walking 🡪 △ visuospatial | 81 | 264 | 171 | .987 | 43,842 | .022 | 5 | .172 |
| 3. visuospatial 🡪 △ walking | 81 | 263 | 171 | .987 | 43,841 | .022 | 6 | .112 |
| Verbal memory and grip strength | |  |  |  |  |  |  |  |
| 1. No coupling | 78 | 562 | 174 | .963 | 53,543 | .045 |  |  |
| **2. grip 🡪 △ memory** | **81** | **487** | **171** | **.970** | **53,489** | **.041** | **75** | **<.001** |
| 3. memory 🡪 △ grip | 81 | 554 | 171 | .964 | 53,556 | .045 | 8 | .046 |
| Processing speed and grip strength | |  |  |  |  |  |  |  |
| 1. No coupling | 87 | 857 | 263 | .957 | 59,960 | .045 |  |  |
| **2. △ grip 🡪 △ processing** | **90** | **814** | **260** | **.960** | **59,937** | **.044** | **43** | **<.001** |
| 3. △ processing 🡪 △ grip | 90 | 848 | 260 | .957 | 59,972 | .046 | 9 | .029 |
| Visuospatial ability and grip strength | |  |  |  |  |  |  |  |
| **1. No coupling** | **78** | **346** | **174** | **.983** | **52,836** | **.030** |  |  |
| 2. △ walking 🡪 △ visuospatial | 81 | 339 | 171 | .984 | 52,850 | .030 | 7 | .072 |
| 3. △ visuospatial 🡪 △ grip | 81 | 335 | 171 | .984 | 52,845 | .030 | 11 | .012 |
| *Note.* Models shown in bold are the best fitting models. | | | | | | | | |

| **Supplementary Table 22.** Changes in Fit of the Bivariate Latent Change Score Models Additionally Including Pathways From Earlier Levels in Physical Function to Upcoming Changes in Cognitive function in each Model. | | | | | | | | |
| --- | --- | --- | --- | --- | --- | --- | --- | --- |
| Model | Parameters | X^2^ | df | CFI | BIC | RMSEA | △X^2^ | *p* |
| Verbal memory and walking time | | | | | | | | |
| **1. No coupling** | **85** | **415** | **167** | **.965** | **44,524** | **.037** |  |  |
| 2. △ walking 🡪 △ memory | 87 | 407 | 165 | .966 | 44,531 | .037 | -8 | .018 |
| 3. △ memory 🡪 walking | 87 | 415 | 165 | .965 | 44,538 | .037 | - |  |
| Processing speed and walking time | |  |  |  |  |  |  |  |
| **1. No coupling** | **94** | **752** | **256** | **.953** | **50,844** | **.042** |  |  |
| 2. △ walking 🡪 △ processing | 96 | 748 | 254 | .953 | 50,854 | .042 | -4 | .135 |
| 3. △ processing 🡪 △ walking | 96 | 749 | 254 | .953 | 50,856 | .042 | -3 | .223 |
| Verbal memory and grip strength | |  |  |  |  |  |  |  |
| **1. No coupling** | **85** | **420** | **167** | **.976** | **53,450** | **.037** |  |  |
| 2. △ grip 🡪 △ memory | 87 | 418 | 165 | .976 | 53,462 | .037 | -2 | .368 |
| 3. △ memory 🡪 △ grip | 87 | 413 | 165 | .976 | 53,457 | .037 | -7 | .030 |
| Processing speed and grip strength | |  |  |  |  |  |  |  |
| 1. No coupling | 94 | 747 | 256 | .964 | 59,898 | .042 |  |  |
| 2. △ grip 🡪 △ processing | 96 | 743 | 254 | .964 | 59,909 | .042 | -4 | .135 |
| **3. △ processing 🡪 △ grip** | **96** | **725** | **254** | **.966** | **59,890** | **.041** | **-22** | **<.001** |
| *Note.* Models shown in bold are the best fitting models. | | | | | | | | |

**References**

1. Wechsler D. *Wechsler Memory Scale III-UK Administration and Scoring Manual*. London; 1998.

2. Wechsler D. *Wechsler Adult Intelligence Scale III-UK Administration and Scoring Manual*. London: Psychological Corporation; 1998.

3. Deary IJ, Simonotto E, Meyer M, et al. The functional anatomy of inspection time: an event-related fMRI study. *Neuroimage*. 2004;22(4):1466–1479. doi:10.1016/j.neuroimage.2004.03.047

4. Deary IJ, Der G, Ford G. Reaction times and intelligence differences: A population-based cohort study. *Intelligence*. 2001;29(5):389–399. doi:https://doi.org/10.1016/S0160-2896(01)00062-9

5. Hamaker EL, Kuiper RM, Grasman RP. A critique of the cross-lagged panel model. *Psychol Methods*. 2015;20(1):102. doi:10.1037/a0038889

6. Ritchie SJ, Tucker-Drob EM, Starr JM, Deary IJ. Do cognitive and physical functions age in concert from age 70 to 76? Evidence from the Lothian Birth Cohort 1936. *Span J Psychol*. 2016;19. doi:10.1017/sjp.2016.85

7. Grimm KJ, Ram N, Estabrook R. *Growth Modeling: Structural Equation and Multilevel Modeling Approaches*. Guilford Publications; 2016.
